# Supplementary material for: Predicted antiviral potential of phytochemicals prolific in Cleistanthus bracteosus Jabl. and essential oils of Artemisia scoparia and Thuja orientalis against Nipah virus and Human metapneumovirus: An AI-driven in-silico study
Source: PLoS One. 2026 Mar 31;21(3):e0346254. doi: 10.1371/journal.pone.0346254 (PMC13038001; doi:10.1371/journal.pone.0346254)
Supplement: S1 Table — (DOCX) [file pone.0346254.s001.docx]

Table S1. The complete GC-MS profile of *C. bracteosus*

| Extract type | Extract fraction | Positive bioactive property of fraction | Compound detected | GC-MS | | |
| --- | --- | --- | --- | --- | --- | --- |
|  |  |  |  | M/Z | RRT | Area % |
| *C. bracteosus* stem | F1 | Synergistic with ampicillin and penicillin G | Hexadecanoic acid, methyl ester | 270 | 8.82 | 2.557 |
|  |  |  | Methyl stearate | 298 | 11.25 | 4.005 |
|  |  |  | Octadecanamide | 283 | 13.19 | 1.779 |
|  | F2 | Synergistic with ampicillin and penicillin G | 4-Methyl-2,4-bis(4'-trimethylsilyloxyphenyl) pentene-1 | 412 | 15.79 | 0.211 |
|  |  |  | Trimethyl [4-(1,1,3,3,-tetramethylbutyl) phenoxy] silane | 278 | 18.69 | 0.351 |
|  | F3 | Synergistic with penicillin G | Benzenedodecanoic acid | 378 | 23.09 | 0.658 |
|  |  |  | Hexadecanoic acid, methyl ester | 270 | 21.68 | 0.864 |
|  | F4 | Synergistic with ampicillin and penicillin G | Hexasiloxane | 431 | 6.92 | 2.973 |
|  |  |  | Octasiloxane | 563 | 10.46 | 1.031 |
|  |  |  | 1-Monolinoleoylglycerol trimethylsilyl ether | 469 | 16.62 | 1.944 |

Table S1 (continued)

| Extract type | Extract fraction | Positive bioactive property of fraction | Compound detected | GC-MS | | |
| --- | --- | --- | --- | --- | --- | --- |
|  |  |  |  | M/Z | RRT | Area % |
|  |  |  | Heptasiloxane, hexadecamethyl | 517 | 8.29 | 1.693 |
|  | F5 | Standalone antibacterial activity and synergistic with penicillin G | 9, 12-Octadecadienoic acid | 280 | 22.93 | 0.301 |
|  |  |  | 1-Monolinoleoylglycerol trimethylsilyl ether | 469 | 17.24 | 0.595 |
|  |  |  | Oleic acid | 282 | 17.4 | 1.320 |
|  | F6 | Standalone antibacterial activity and synergistic with penicillin G | Hexadecanoic acid, methyl ester | 270 | 22.61 | 1.250 |
|  |  |  | 1-Monolinoleoylglycerol trimethylsilyl ether | 469 | 17.04 | 1.396 |
|  | F7 | Standalone antibacterial activity and synergistic with penicillin G | Hexadecanoic acid, methyl ester | 270 | 22.94 | 2.608 |
|  |  |  | 9,12,15-Octadecatrienoic acid | 443 | 22.92 | 2.608 |

Table S1 (continued)

| Extract type | Extract fraction | Positive bioactive property of fraction | Compound detected | GC-MS | | |
| --- | --- | --- | --- | --- | --- | --- |
|  |  |  |  | M/Z | RRT | Area % |
|  | F8 | Standalone antibacterial activity and synergistic with penicillin G | 7aH-Cyclopenta(a)cyclopropa(f)cycloundecene-2,4,7,7a,10,11-hexol,1,1a,2,3,4,4a | 580 | 14.42 | 0.883 |
|  |  |  | Hexadecanoic acid, methyl ester | 270 | 14.44 | 0.587 |
|  | F9 | Synergistic with penicillin G | Hexadecanoic acid, methyl ester | 270 | 16.25 | 1.973 |
| *C. bracteosus* bark | F1 | Standalone antibacterial activity | 4-Methyl-2,4-bis(4'-trimethylsilyloxyphenyl) pentene-1 | 412 | 18.87 | 1.212 |

Table S1 (continued)

| Extract type | Extract fraction | Positive bioactive property of fraction | Compound detected | GC-MS | | |
| --- | --- | --- | --- | --- | --- | --- |
|  |  |  |  | M/Z | RRT | Area % |
|  | F2 | Standalone antibacterial activity | Oleic acid | 282 | 12.44 | 0.815 |
|  | F3 | Standalone antibacterial activity | Oleic acid | 282 | 23.04 | 0.638 |
|  |  |  | Acevaltrate | 480 | 16.03 | 0.536 |
|  | F4 | Synergistic with ampicillin and penicillin G | α-Asarone | 209 | 17.70 | 0.354 |
|  |  |  | 1-Monolinoleoylglycerol trimethylsilyl ether | 469 | 13.68 | 0.705 |
|  | F5 | Standalone antibacterial activity | 1-Gala-1-ido-octose | 240 | 14.46 | 0.541 |

Table S1 (continued)

| Extract type | Extract fraction | Positive bioactive property of fraction | Compound detected | GC-MS | | |
| --- | --- | --- | --- | --- | --- | --- |
|  |  |  |  | M/Z | RRT | Area % |
|  | F6 | Standalone antibacterial activity and synergistic with ampicillin and penicillin G | Digoxigenin | 388 | 2.68 | 4.176 |
|  |  |  | Lauric acid or dodecanoic acid | 200 | 9.32 | 0.769 |
|  | F7 | - | Lauric acid or dodecanoic acid | 200 | 16.26 | 2.167 |
| *C. bracteosus* wood | F1 | - | β-D-Glucopyranosiduronic acid | 228 | 22 | 0.558 |
|  | F2 | - | Tetradecanoic acid | 326 | 18.96 | 0.691 |
|  | F3 | - | Gibberellic acid | 346 | 2.81 | 0.679 |
|  |  |  | Acetic acid | 60 | 7.6 | 0.322 |
|  | F4 | Standalone antibacterial activity and synergistic with penicillin G | Stearic acid | 594 | 18.07 | 0.365 |

Table S1 (continued)

| Extract type | Extract fraction | Positive bioactive property of fraction | Compound detected | GC-MS | | |
| --- | --- | --- | --- | --- | --- | --- |
|  |  |  |  | M/Z | RRT | Area % |
|  | F5 | - | 4-Methyl-2,4-bis(4'-trimethylsilyloxyphenyl) pentene-1 | 412 | 13.19 | 0.263 |
|  |  |  | Trimethyl [4-(1,1,3,3,-tetramethylbutyl) phenoxy] silane | 278 | 22.03 | 0.207 |
|  | F6 | - | 9, 12-Octadecadienoic acid | 443 | 20.68 | 0.486 |
|  | F7 | - | Heptacosane | 380 | 13.62 | 0.088 |
|  |  |  | Octadecane 3-ethyl-5-(2-ethylbutyl)- | 366 | 15.31 | 2.277 |

F = Isolated fraction of extract, M/Z = Mass-to-charge ratio, RRT = Relative retention time.
