# Supplementary material for: Predicted antiviral potential of phytochemicals prolific in Cleistanthus bracteosus Jabl. and essential oils of Artemisia scoparia and Thuja orientalis against Nipah virus and Human metapneumovirus: An AI-driven in-silico study
Source: PLoS One. 2026 Mar 31;21(3):e0346254. doi: 10.1371/journal.pone.0346254 (PMC13038001; doi:10.1371/journal.pone.0346254)
Supplement: S2 Table — (DOCX) [file pone.0346254.s002.docx]

Table S2. The complete GC-MS profile of *A. scoparia* and *T. orientalis*

| Extract type | Positive bioactive property of extract | Compound detected | GC-MS | | |
| --- | --- | --- | --- | --- | --- |
|  |  |  | M/Z | RRT | Area % |
| *A. scorparia* EO | Antibacterial and antifungal | α-Pinene | 136 | 7.73 | 0.479 |
|  |  | β- Pinene | 136 | 9.22 | 1.507 |
|  |  | α-Myrcene | 136 | 10.24 | 1.007 |
|  |  | γ-Terpinene | 136 | 10.27 | 1.007 |
|  |  | o-Cymene | 134 | 10.34 | 4.762 |
|  |  | β-Ocimene | 134 | 11.65 | 1.003 |
|  |  | 1,3-Cyclohexadiene | 80 | 18.34 | 6.283 |
|  |  | Bornyl acetate | 196 | 20.06 | 0.396 |
|  |  | Epiglobulol | 222 | 21.43 | 0.823 |
|  |  | Thujopsene-(I3) | 204 | 22.06 | 0.423 |
|  |  | Caryophyllene | 204 | 24.59 | 2.696 |
|  |  | γ-Elemene | 204 | 25.68 | 1.577 |

Table S2 (continued)

| Extract type | Positive bioactive property of extract | Compound detected | GC-MS | | |
| --- | --- | --- | --- | --- | --- |
|  |  |  | M/Z | RRT | Area % |
|  |  | β-Copaene | 204 | 26.5 | 1.577 |
|  |  | cis-β-Farnesene | 204 | 26.7 | 0.654 |
|  |  | Cedrene | 204 | 26.7 | 11.627 |
|  |  | β-Copaene | 204 | 27.6 | 0.748 |
|  |  | (E)-β-Famesene | 204 | 27.74 | 0.466 |
|  |  | (-)-Tau-muurolol | 222 | 31.6 | 1.048 |
|  |  | α-Cadinol | 222 | 31.6 | 0.987 |
|  |  | 1-Heptatriacotanol | 537 | 37.67 | 0.380 |
|  |  | Palmitoleic acid | 254 | 37.75 | 1.167 |
|  |  | Pentadecanoic acid | 242 | 38.29 | 1.756 |
|  |  | 1-Heptatriacotanol | 537 | 40.47 | 1.183 |

Table S2 (continued)

| Extract type | Positive bioactive property of extract | Compound detected | Compound detected | | |
| --- | --- | --- | --- | --- | --- |
|  |  |  | M/Z | RRT | Area % |
|  |  | Podocarp-7-en-3-ol, 13-methyl-13-vinyl | 288 | 41.47 | 1.183 |
|  |  |  |  | 41.84 | 2.103 |
| *T. orientalis* EO | Antibacterial and antifungal | Geranyl-α-terpinene | 272 | 45.35 | 0.746 |
|  |  | 2-Carene | 136 | 3.42 | 0.553 |
|  |  | α-Terpineol | 137 | 4.46 | 0.629 |
|  |  | Bornyl acetate | 196 | 5.33 | 0.869 |
|  |  | Caryophyllene | 204 | 6.71 | 5.597 |
|  |  | cis-Thujopsene | 204 | 7.23 | 2.300 |
|  |  | Cubenol | 222 | 7.75 | 1.646 |
|  |  | Ledol | 222 | 8.26 | 0.906 |
|  |  | Cedrol | 222 | 8.4 | 13.487 |
|  |  | α-Cardinol | 222 | 8.66 | 3.017 |

Table S2 (continued)

| Extract type | Positive bioactive property of extract | Compound detected | Compound detected | | |
| --- | --- | --- | --- | --- | --- |
|  |  |  | M/Z | RRT | Area % |
|  |  | Guaiol | 222 | 8.69 | 2.020 |
|  |  | Epiglobulol | 222 | 8.84 | 0.333 |
|  |  | Thujopsene-(I3) | 204 | 9.51 | 1.479 |
|  |  | n-Hexadecanoic acid | 256 | 10.7 | 0.957 |
|  |  | Podocarp-7-en-3-ol, 13-methyl-13-vinyl | 288 | 12.85 | 7.017 |

M/Z = Mass-to-charge ratio, RRT = Relative retention time.
