# Supplementary material for: Predicted antiviral potential of phytochemicals prolific in Cleistanthus bracteosus Jabl. and essential oils of Artemisia scoparia and Thuja orientalis against Nipah virus and Human metapneumovirus: An AI-driven in-silico study
Source: PLoS One. 2026 Mar 31;21(3):e0346254. doi: 10.1371/journal.pone.0346254 (PMC13038001; doi:10.1371/journal.pone.0346254)
Supplement: S1 Fig — (PDF) [file pone.0346254.s003.pdf]

PROCHECK

2VWD (a)

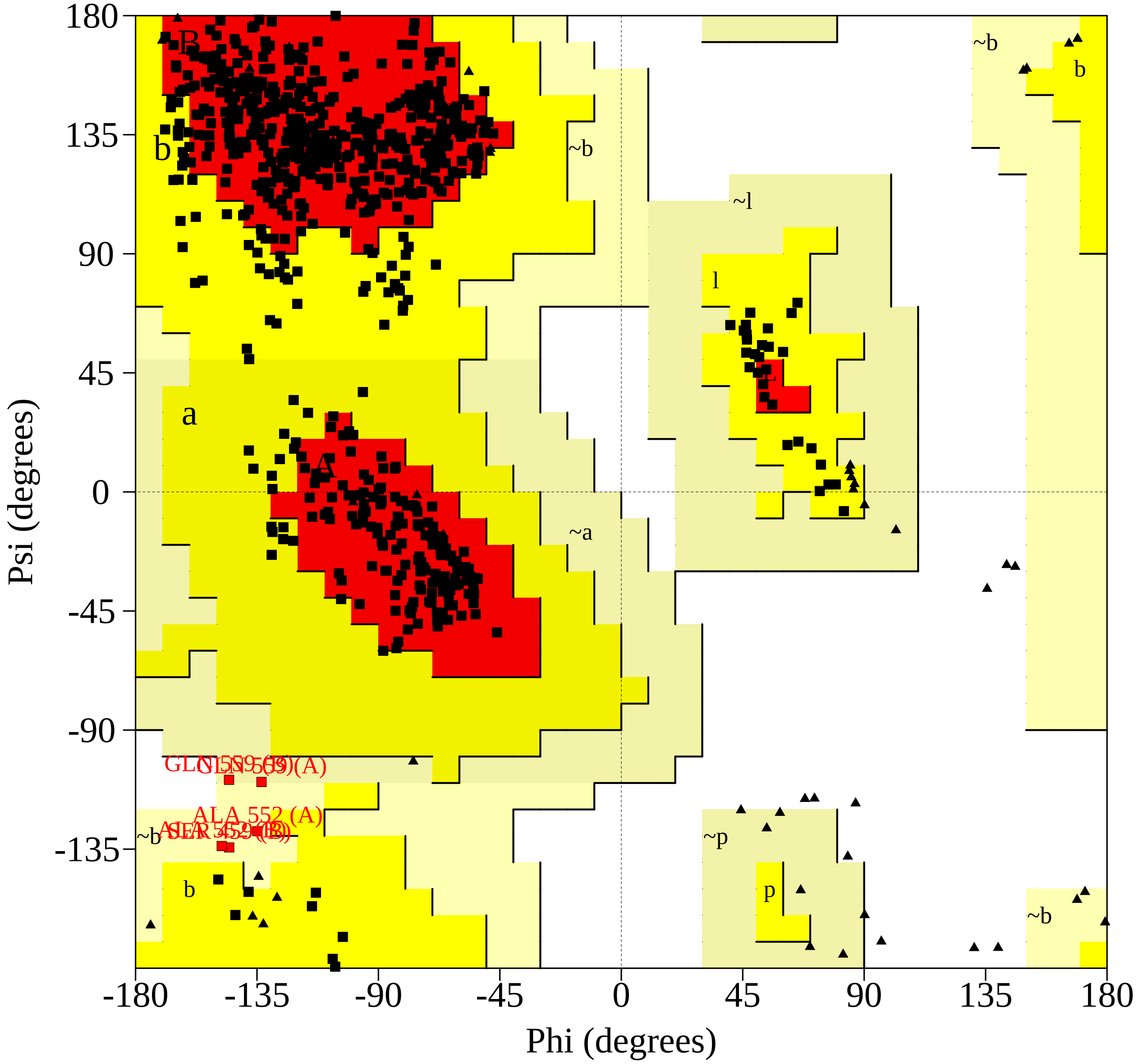

Plot statistics

Residues in most favoured regions [A,B,L]	597	84.0%
Residues in additional allowed regions [a,b,l,p]	109	15.3%
Residues in generously allowed regions [~a,~b,~l,~p]	5	0.7%
Residues in disallowed regions	0	0.0%
	----	------
Number of non-glycine and non-proline residues	711	100.0%
Number of end-residues (excl. Gly and Pro)	18	
Number of glycine residues (shown as triangles)	54	
Number of proline residues	50	

Total number of residues	833	

Based on an analysis of 118 structures of resolution of at least 2.0 Angstroms and R-factor no greater than 20%, a good quality model would be expected to have over 90% in the most favoured regions.

PROCHECK

5EVM (b)

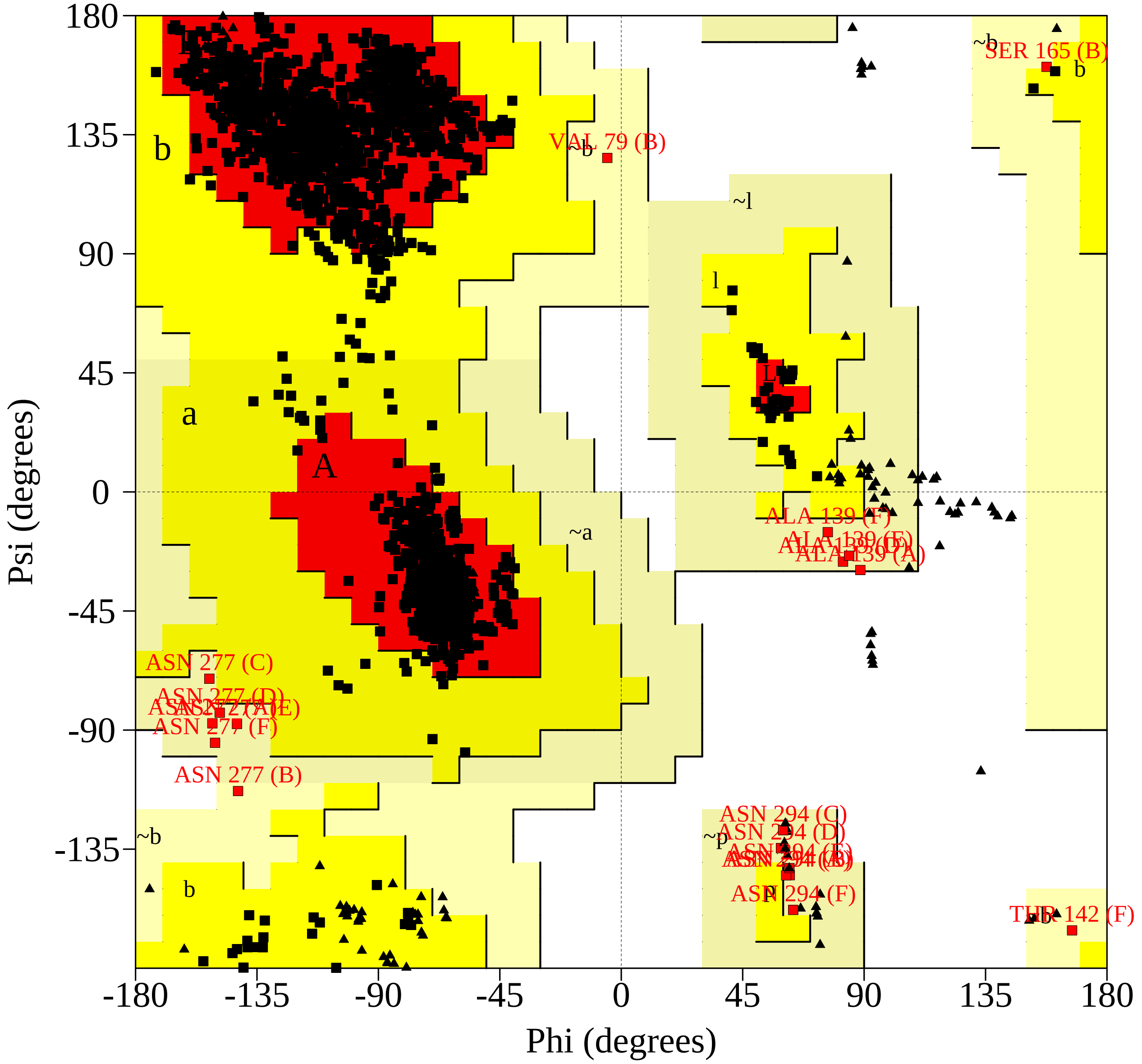

Plot statistics

Residues in most favoured regions [A,B,L]	2324	93.6%
Residues in additional allowed regions [a,b,l,p]	141	5.7%
Residues in generously allowed regions [~a,~b,~l,~p]	19	0.8%
Residues in disallowed regions	0	0.0%
	----	------
Number of non-glycine and non-proline residues	2484	100.0%
Number of end-residues (excl. Gly and Pro)	12	
Number of glycine residues (shown as triangles)	150	
Number of proline residues	90	

Total number of residues	2736	

Based on an analysis of 118 structures of resolution of at least 2.0 Angstroms and R-factor no greater than 20%, a good quality model would be expected to have over 90% in the most favoured regions.

PROCHECK

5WBO (c)

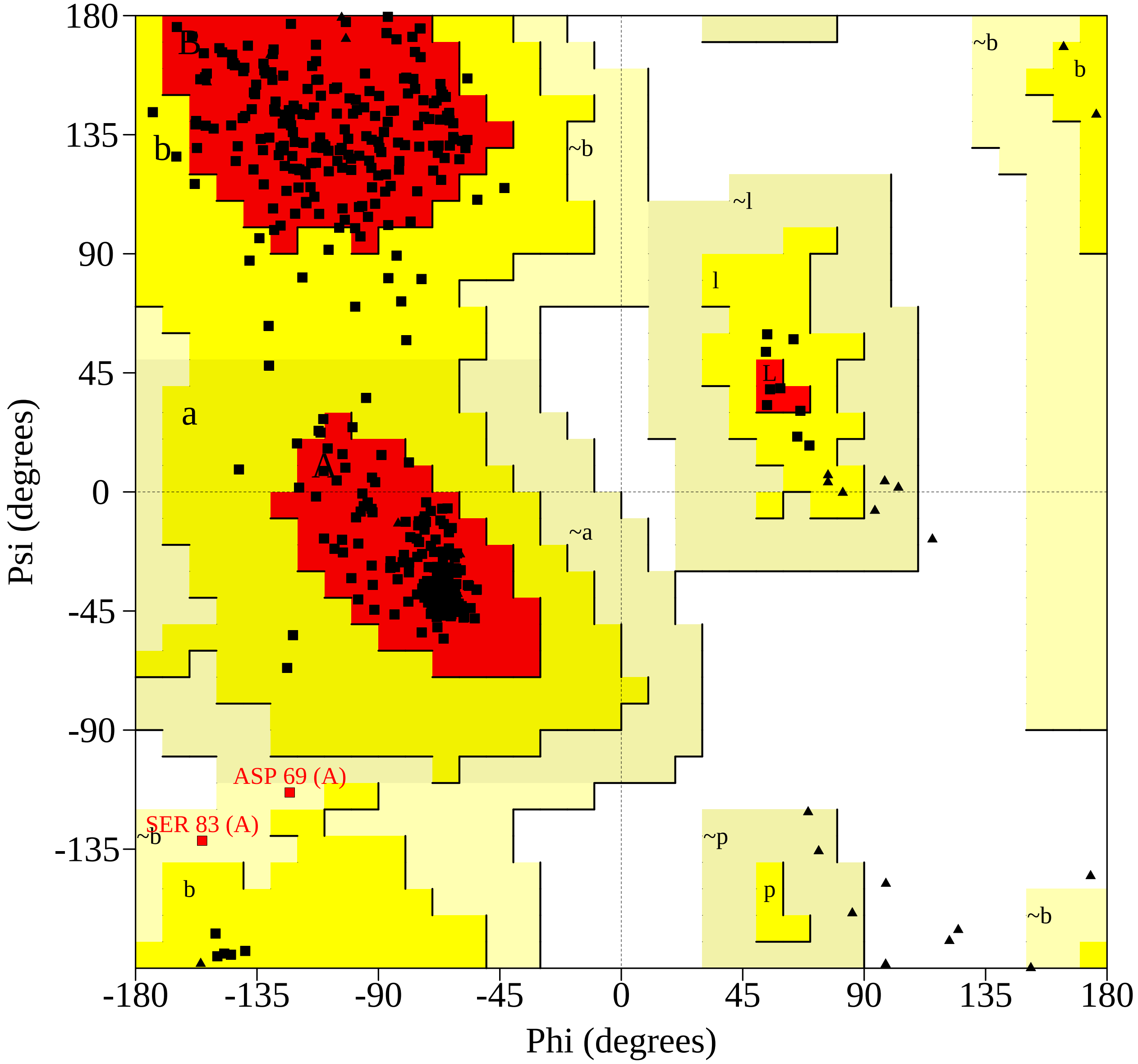

Plot statistics

Residues in most favoured regions [A,B,L]	370	90.5%
Residues in additional allowed regions [a,b,l,p]	37	9.0%
Residues in generously allowed regions [~a,~b,~l,~p]	2	0.5%
Residues in disallowed regions	0	0.0%
	----	------
Number of non-glycine and non-proline residues	409	100.0%
Number of end-residues (excl. Gly and Pro)	7	
Number of glycine residues (shown as triangles)	29	
Number of proline residues	14	

Total number of residues	459	

Based on an analysis of 118 structures of resolution of at least 2.0 Angstroms and R-factor no greater than 20%, a good quality model would be expected to have over 90% in the most favoured regions.

llu7_01.ps
