# Supplementary material for: Predicted antiviral potential of phytochemicals prolific in Cleistanthus bracteosus Jabl. and essential oils of Artemisia scoparia and Thuja orientalis against Nipah virus and Human metapneumovirus: An AI-driven in-silico study
Source: PLoS One. 2026 Mar 31;21(3):e0346254. doi: 10.1371/journal.pone.0346254 (PMC13038001; doi:10.1371/journal.pone.0346254)
Supplement: Attachment — Submitted filename: reviewed_PONE-D-25-23763.docx [file pone.0346254.s004.docx]

Major secondary metabolites present in Cleistanthus bracteosus Jabl. and essential

oils of Artemisia scoparia and Thuja orientalis have anti-Nipah virus and anti-human

metapneumovirus potential: An AI-driven in-silico study

Manuscript Number: **PONE-D-25-23763**

Dear Editor,

The authors have carried out experiments to identify the chemical constituents of different plants and then explored its potential for therapeutic values against different viruses using in silico tools. The work carries some scientific merit and following points were deduced

Major points-

1. The docking scores of major phytochemicals have been compared to that of chloroquine. However, the specificity of this drug with the selected target proteins have not been mentioned. Any experimental evidence (literature citation) would support this claim. In other words, would chloroquine bind to proteins with PDB ID of 2VWD, 5EVM, and 5WB0 and not with others of the virus?
2. The RMSD for 3 cases (proteins) have been reported. It is not clear if it is ligand RMSD or protein backbone RMSD. The least square fitting for the reference geometry is not mentioned. Two types of curves (one for ligand RMSD and another for protein backbone RMSD both relative to protein backbone) for each protein-ligand adduct are required for proper analysis of molecular dynamics trajectories. Also, the authors have stated 3 runs (triplicate) and no corresponding data (standard deviation) could be found.
3. The RMSD and RMSF profiles of multiple chains have been presented. Please provide the relevance of these. Any basis for comparative study is not found. What was seen before docking and what was obtained after ligand binding? This stands as one of the prime metrics to be resolved.
4. Does the ligand (phytochemicals and chloroquine) stay at the binding pocket at 100 ns? Please provide pictorial evidence of it for the different cases. This would strengthen the results of simulation.
5. The significance of the output needs to be provided thoroughly than just stating the numbers. Adequate comparison to the existing literature would be better.
6. The GC-MS plots are not readable. Multiple peaks suggest many molecules. Consideration of large dataset of phytochemicals would be better as computational work.

Other points-

1. Language/grammar requires a good fixation. An acceptable scientific writing would be appropriate. (2^nd^ sentence of abstract, line 66, line 68…). Typographical errors are present and require thorough correction. (Eg: Nipa virus)
2. The figures are not clear and the texts/numbers are not readable. High resolution image (vector graphics preferably) would help to understand the outcomes.
3. RMSD= root mean square deviation
4. Line 71: “Furthermore, the RMSD and RMSF graphs show fluctuations at various points in the protein-ligand complex system due to structural changes in the beta-turn-beta and helix-coil-helix alterations at specific points resulting in increased RMSD and RMSF within a time frame of 50 ns.” How is it known that beta-turn-beta and helix-coil-helix are involved? Please provide the data to support it? Also, the time mentioned could not be comprehended.
5. “Outbreaks occur almost annually in Bangladesh, and studies have linked infections to drinking fermented date juice contaminated with urine from bats.” Please provide a reference.
6. Sentence starting at Line 159.
7. The criteria for the selection of *Cleistanthus bracteosus* Jabl., *Artemisia scoparia*, and *Thuja orientalis* are missing in the introduction part. This is a crucial point as the whole research thesis is dependent on it.
8. Please incorporate past-tense in the methodology part.
9. “The rationality for selecting the aforementioned phytochemical compounds include their high abundance in the selected plant extracts and their prominence of inducing a variety of broad-spectrum antimicrobial activities.” The chemical space of 4 molecules is relatively small considering large number of phytochemicals present in the three plants. What is the abundance as the authors have mentioned it to be high? Reference for broad-spectrum properties (especially anti-viral) would help.
10. The type of charges for the ligands is missing.
11. The convergence studies of the control parameters for molecular docking calculations have not been presented. It would add value to the final docking scores.
12. How many poses were generated for each ligand?
13. How were the active sites of the proteins selected? No information has been presented. It is important to include the details. Was blind docking performed? Please add relevant references.
14. Line 284. Please elaborate about accurate output. How is it assessed or what was the basis?
15. How was the cut-off of ≤-6 kcal/mol chosen? Please provide references.
16. Line 297- “which assisted in more insight into protein and docked complexes under biological conditions” Please clarify this part of the sentence. 300 K is not human biological condition.
17. Line 313. Please elaborate about Gibbs ensemble.
18. Lines 328-331. Please edit it.
19. Please provide the significance of “Physicochemical properties of NiV and hMPV proteins”. Why is it necessary? (Table 1)
20. “The low value of GRAVY for the selected receptors of NiV and hMPV suggests significant interactions with water molecules…” The molecular docking was not carried out in a hydrated environment. Please clarify.
21. “Using Autodock Vina software, the cut-off value for predicting high docking energy between a ligand and a receptor has been set at −6 kcal/mol in previous studies [51, 52].” The value is not obvious in reference 51. In reference 52, the name of journal is missing. Still the statistical significance is not discussed.
22. The key amino acid residues in case of F0 receptor are all different for 3 ligands. How do these compare with that of chloroquine? Does the dissimilarity point to the same pocket? Please provide additional interpretation.
23. The same point as in number 21 applies to another receptor G.
24. The scores for chloroquine are not better than -6 kcal/mol in two cases. Please comment.
25. LYS-254 is the only common one in case of hMPV. Please compare with those with chloroquine. The type of interaction is not disclosed.
26. Chloroquine showed AMES toxicity, hepatotoxicity, hERG II inhibition, and low HIA. It was predicted to be a substrate for 4 instances. The ADMET prediction was not suitable for it to be a safe drug. Please clarify.
27. “RMSD and RMSF values of protein-ligand complexes were compared to identify any minor or major alterations in the structural configuration of viral protein receptor after ligand binding.” Compared with which other case? The results before ligand binding are not reported.
28. “Lower RMSD value has been proposed as an indicator of more stable docked configuration [56].” This reference discusses about crystal structures with good resolution. The proteins in this study have high values. Please correlate the current case with that mentioned in the reference.
29. Table 5 may be better with the results of chloroquine for better comparison. The use of multiple chains is not clear. Please clarify and provide adequate references.
30. The calculation of the apo form of the proteins for relative stability analysis would help readers to assess the consequences of adduct formation. A significant point seems to be missing.
31. Please adjust the significant figures for distances.
32. “In case of 2VWD, all ligands molecules provide protein-like stability.” Please elaborate.
33. “Subsequently, minor fluctuations between 250 and 300 residues were observed for 5WB0 and its ligand complexes” Where are these residues located and are there any disruption to ligand binding?
34. “Whereas chloroquine is one of the very few potential agents that has been proposed as an option against preventing the host-cell attachment and entry of NiV [66]” This reference does not include chloroquine. Please provide an adequate one. Also, the author names are missing in the list.
35. “We selected four compounds of plant origin for which data in the literature have demonstrated antiviral properties against different viruses”. Please add the references.
36. Lines 502 and 503 are broken. Please fix it. It is better in introduction section where a case is built.
37. Relevance of reference 73. Please elaborate.
38. Lines 504-523. If the ligands have been reported to possess antiviral properties in the literature, then in silico could be carried out directly for theoretical assessment. The source may be optional. The objective of exploration of natural product-based formulation from the 3 plants chosen could be mentioned.
39. “ADME and toxicity studies of chloroquine, digoxigenin, cedrene and cedrol has been previously reported with slight variations in analysis parameters” What are the slight variations and are they relevant?
40. “MD simulations showed that when the drug receptor complex's structure changed over time, it stabilized in the physiochemical environment.” How is this claim derived?
41. Please avoid the phrase virtual screening since only 4 phytochemicals were used. Also, it is not ADMET-AI. Please omit it (AI) from keywords and abstract.
42. Optional. Other parameters like gyradius, SASA, and radial pair distribution function would help to better assess the level of adduct stability.

Considering all the points, it seems obvious that the manuscript is not suitable for publication in its current form. A major revision with correct, clear, and acceptable answers would likely render it for the next stage.

Thank you.

Sincerely,

Reviewer
