# Supplementary material for: Predicted antiviral potential of phytochemicals prolific in Cleistanthus bracteosus Jabl. and essential oils of Artemisia scoparia and Thuja orientalis against Nipah virus and Human metapneumovirus: An AI-driven in-silico study
Source: PLoS One. 2026 Mar 31;21(3):e0346254. doi: 10.1371/journal.pone.0346254 (PMC13038001; doi:10.1371/journal.pone.0346254)
Supplement: Attachment — Submitted filename: Reviewers Comment for PONE-D-25-23763.docx [file pone.0346254.s005.docx]

**Reviewer’s Comment**

**Manuscript Number: PONE-D-25-23763**

**Title:** Major secondary metabolites present in Cleistanthus bracteosus Jabl. and essential oils of Artemisia scoparia and Thuja orientalis have anti-Nipah virus and anti-human metapneumovirus potential: An AI-driven in-silico study

**Review Summary**

This study deals with the investigation of anti-Nipah virus and anti-human metapneumovirus potential: of major secondary metabolites present in Cleistanthus bracteosus Jabl. and essential oils of Artemisia scoparia and Thuja orientalis. This present study offers computational insights into the nti-Nipah virus and anti-human metapneumovirus potential of these metabolites by using an AI-driven in silico approach. The binding affinities further revealed that some of these compounds interacted more strongly with NiV and hMPV receptors than the drug control chloroquine (-5.5 to -6.1 kcal/mol). Moreover, MD simulations illustrated phytochemical interacting amino acid residues associated with each receptor of NiV and hMPV. These phytochemical compounds were further evaluated using ADMET-AI platforms. The research is relevant, however, several aspects of the study, particularly in terms of methodological clarity and scientific rigor, require significant improvement for the manuscript to meet publication standards.

- The keywords are too long and too many. It is recommended to limit the keywords between four to six
- Line 70, there should be a comma after the word “particular”
- Below line 186 under the materials and method, authors should comment on the grade of reagents and solvents used or report on the purity level of these reagents and solvents
- Line 215, kindly justify why extraction temperature as high as 100^o^C was used knowing fully well that most of the bioactive metabolites are heat-labile
- Line 219, the description of the rotary evaporation should the phrase “under reduced pressure”
- Line 494: put the expression “in vitro” in italics
- Line 543, the word activity should be in plural form to become “anti-NiV and anti-hMPV activities”
- Line544, there should be a comma after the word “particularly”
- Line 778 Ref 67: put the expression “in vivo” in italics
- Line 789 Ref 71: put the expression “in vitro” in italics
- Line 826 Ref 84: put the expression “in vitro” in italics
- Line 606 Ref 2, the article title should be in lower case to become “Pandemics throughout history”
- Line 617 Ref 8, the article title should be in lower case to become “Virus transmission from bats to humans associated with drinking traditional liquor made from Date Palm Sap, Bangladesh”
- Line 675 Ref 28, kindly put the name “Cleistanthus collinus” in italics being a botanical name.
- Line 677 Ref 29: kindly put the name “Artemisia scoparia” in italics being a botanical name.
- Line 683 Ref 32: the article title should be in lower case to become “Phytochemical 684 profiling, antioxidant, antimicrobial and cholinesterase inhibitory effects of essential 685 oils isolated from the leaves of Artemisia scoparia and Artemisia absinthium
- Line 685 Ref 32: kindly put the names “Artemisia scoparia” and “Artemisia absinthium” in italics being botanical names.
