# Supplementary material for: Predicted antiviral potential of phytochemicals prolific in Cleistanthus bracteosus Jabl. and essential oils of Artemisia scoparia and Thuja orientalis against Nipah virus and Human metapneumovirus: An AI-driven in-silico study
Source: PLoS One. 2026 Mar 31;21(3):e0346254. doi: 10.1371/journal.pone.0346254 (PMC13038001; doi:10.1371/journal.pone.0346254)
Supplement: Attachment — Submitted filename: PLOS One rebuttal and response to reviewers.docx [file pone.0346254.s006.docx]

**REVIEWER 1 COMMENTS AND RESPONSES**

**Main response from first author:**

Thank you for your valuable comments which helped us to make significant improvements to this manuscript. We have made the suggested amendments that you have requested where possible.

All suggested revisions and corrections made according to Reviewer 1 have been highlighted in red colour in the manuscript.

In case where all reviewers have made the same/similar request/s, those responses have

been highlighted in teal colour in the manuscript.

Major secondary metabolites present in Cleistanthus bracteosus Jabl. and essential

oils of Artemisia scoparia and Thuja orientalis have anti-Nipah virus and anti-human

metapneumovirus potential: An AI-driven in-silico study

Manuscript Number: PONE-D-25-23763

Dear Editor,

The authors have carried out experiments to identify the chemical constituents of different plants and then explored its potential for therapeutic values against different viruses using in silico tools. The work carries some scientific merit and following points were deduced

Major points-

1.	The docking scores of major phytochemicals have been compared to that of chloroquine. However, the specificity of this drug with the selected target proteins have not been mentioned. Any experimental evidence (literature citation) would support this claim. In other words, would chloroquine bind to proteins with PDB ID of 2VWD, 5EVM, and 5WB0 and not with others of the virus?

**Response from author:** Research utilizing animal models has also investigated the potential application of chloroquine against NiV and hMPV, where chloroquine has been used as an antagonist in preventing the host-cell attachment and entry of these two viruses. However, currently there is no standard antiviral therapy for these viral infections. Additional references have been added to justify this statement.

2.	The RMSD for 3 cases (proteins) have been reported. It is not clear if it is ligand RMSD or protein backbone RMSD. The least square fitting for the reference geometry is not mentioned. Two types of curves (one for ligand RMSD and another for protein backbone RMSD both relative to protein backbone) for each protein-ligand adduct are required for proper analysis of molecular dynamics trajectories. Also, the authors have stated 3 runs (triplicate) and no corresponding data (standard deviation) could be found.

**Response from author:** The protein backbone or "Apo protein simulation has been added and “three independent” has been removed.

3.	The RMSD and RMSF profiles of multiple chains have been presented. Please provide the relevance of these. Any basis for comparative study is not found. What was seen before docking and what was obtained after ligand binding? This stands as one of the prime metrics to

be resolved.

**Response from author:** NiV attachment glycoprotein (G) (PDB ID: 2VWD) consist of two chains in its protein structure file (Chain A and Chain B), Meanwhile, the NiV fusion glycoprotein (F0) (PDB ID: 5EVM) has six chains in its protein structure file (Chain A, Chain B, Chain C, Chain D, Chain E and Chain F). In which, they are differ from each other. All these chains individually interact with the selected ligand upon docking with per-residue analysis and therefore, it is important to show their MD simulations.

4.	Does the ligand (phytochemicals and chloroquine) stay at the binding pocket at 100 ns? Please provide pictorial evidence of it for the different cases. This would strengthen the results of simulation.

**Response from author:** Yes, they do except for chloroquine. Binding free energy (MMPBSA) data has been added to validate this.

5.	The significance of the output needs to be provided thoroughly than just stating the numbers. Adequate comparison to the existing literature would be better.

**Response from author**: There are no existing literature for these viruses on treatment options or experimental studies. However, we have provided assumptions and examples from similar studies.

6.	The GC-MS plots are not readable. Multiple peaks suggest many molecules. Consideration of large dataset of phytochemicals would be better as computational work.

**Response from author**: GC-MS data were gathered from our primary research based on these three plants. Multiple peaks consist of artefacts, which has been carefully excluded upon individually checking with the computerised MS–data bank NIST (National Institute of Standards and Technology) 2.0 (2005) database. You may refer to the supplementary table added that represents the full GC-MS data of *C. bracteosus* chloroform purified fractions (Table S1) and *A. scorparia* and *T. orientalis* (Table S2) for more details.

Furthermore, kindly refer to the uploaded images (PNG files) in the journal platform, which are of high resolution and not the images present in the converted PDF. Images are needed to be directly added to the PDF upon acceptance for publication.

Other points-

1.	Language/grammar requires a good fixation. An acceptable scientific writing would be appropriate. (2nd sentence of abstract, line 66, line 68…). Typographical errors are present and require thorough correction. (Eg: Nipa virus)

**Response from author:** Revised as suggested.

2.	The figures are not clear and the texts/numbers are not readable. High resolution image (vector graphics preferably) would help to understand the outcomes.

**Response from author**: kindly refer to the uploaded images (PNG files) in the journal platform, which are of high resolution and not the images present in the converted PDF. Images are needed to be directly added to the PDF upon acceptance for publication.

3.	RMSD= root mean square deviation

**Response from author:** Revised as suggested.

4.	Line 71: “Furthermore, the RMSD and RMSF graphs show fluctuations at various points in the protein-ligand complex system due to structural changes in the beta-turn-beta and helix-coil-helix alterations at specific points resulting in increased RMSD and RMSF within a time frame of 50 ns.” How is it known that beta-turn-beta and helix-coil-helix are involved? Please provide the data to support it? Also, the time mentioned could not be comprehended.

**Response from author**: removed RMSF from the sentence. RMSD fluctuations are more obvious up to 50 ns and seemingly getting stable between 50 ns and 100 ns. Structural alterations usually occur in beta-turn-beta and helix-coil-helix in proteins. It is a generalized statement (reference added).

5.	“Outbreaks occur almost annually in Bangladesh, and studies have linked infections to drinking fermented date juice contaminated with urine from bats.” Please provide a reference.

**Response from author:** Revised as suggested. Reference added.

6.	Sentence starting at Line 159.

**Response from author:** Revised as suggested.

7.	The criteria for the selection of Cleistanthus bracteosus Jabl., Artemisia scoparia, and Thuja orientalis are missing in the introduction part. This is a crucial point as the whole research thesis is dependent on it.

**Response from author:** Revised as suggested. More details of similar prior antiviral studies *C. bracteosus*, *A. scorparia* and *T. orientalis* and their potential use as therapeutic agents in folklore medicine to treat viral like infections has been added. However, direct studies involving these three plants against NiV and hMPV has not yet been published. Hence, this can be considered as a pioneering study to show the significance of antiviral potential against via NiV and hMPV *in* *silico* methods. These three plant specimens were a part of our primary research focusing on antimicrobial and synergistic activities.

8.	Please incorporate past-tense in the methodology part.

**Response from author:** Revised as suggested.

9.	“The rationality for selecting the aforementioned phytochemical compounds include their high abundance in the selected plant extracts and their prominence of inducing a variety of broad-spectrum antimicrobial activities.” The chemical space of 4 molecules is relatively small considering large number of phytochemicals present in the three plants. What is the abundance as the authors have mentioned it to be high? Reference for broad-spectrum properties (especially anti-viral) would help.

**Response from author**: Digoxigenin and lauric acid were the only phytochemical compounds detected in the most bioactive TLC isolated fraction of *C. bracteosus*. Unlike cedrene from *A. scorparia* and cedrol from *T. orientalis*, they were not selected based on abundance. However, the total area percentage for lauric acid is 2.936% (0.769% from bark fraction 6 plus 2.167% from bark fraction 7). You may refer to the supplementary table added that represents the full GC-MS data of *C. bracteosus* chloroform purified fractions (Table S1) and *A. scorparia* and *T. orientalis* (Table S2) for more details.

Furthermore, all the available literature related to these three plants and their compounds related to antiviral activity has been already cited.

10.	The type of charges for the ligands is missing.

**Response from author**: type of charges was mentioned under modelling and preparation of selected macromolecules. “Kollman charges were added”.

11.	The convergence studies of the control parameters for molecular docking calculations have not been presented. It would add value to the final docking scores.

**Response from author:** Revised as suggested. Docking values and MD simulations for the controlled drug chloroquine has been added.

12.	How many poses were generated for each ligand?

**Response from author:** molecular dynamics, it was performed with a collection rate of 2 x 10^-15^ (2 fs), which is the classic collection time according to all the literature (newly added to MD simulation section at methods).

13.	How were the active sites of the proteins selected? No information has been presented. It is important to include the details. Was blind docking performed? Please add relevant references.

**Response from author**: Yes, it was blind docking. “Blind docking” has been added at the methods section.

14.	Line 284. Please elaborate about accurate output. How is it assessed or what was the basis?

**Response from author**: We followed the standard protocol for AutoDock Vina combined with MD simulation data along with available literate on molecular docking to assess the outputs.

15.	How was the cut-off of ≤-6 kcal/mol chosen? Please provide references.

**Response from author**: Reference has been already added.

16.	Line 297- “which assisted in more insight into protein and docked complexes under biological conditions” Please clarify this part of the sentence. 300 K is not human biological condition.

**Response from author**: The sentence has been improved and revised. 300 K is an operational condition in GROMACS for performing MD simulations.

17.	Line 313. Please elaborate about Gibbs ensemble.

**Response from author**: this is a widely accepted theory of thermodynamics, over 100 years old. It states that a reaction or state has its favourable configuration if the combined enthalpy and entropy changes produce a negative value, so the final state is a favourable state under normal conditions. The program that calculated it was cited (it's MMPBSA). Also refer to citation 54.

18.	Lines 328-331. Please edit it.

**Response from author:** Revised as suggested.

19.	Please provide the significance of “Physicochemical properties of NiV and hMPV proteins”. Why is it necessary? (Table 1)

**Response from author:** Physicochemical properties of proteins are crucial in drug discovery as they significantly influence a drug's efficacy, safety, and overall behaviour. In short, they determine the protein stability for docking (line).

20.	“The low value of GRAVY for the selected receptors of NiV and hMPV suggests significant interactions with water molecules…” The molecular docking was not carried out in a hydrated environment. Please clarify.

**Response from author:** This refers to the water molecules present within the prtiens and not the testing environment. Water molecules are usually removed, and hydrogen bonds are add prior to docking to elevate efficiency.

21.	“Using Autodock Vina software, the cut-off value for predicting high docking energy between a ligand and a receptor has been set at −6 kcal/mol in previous studies [51, 52].” The value is not obvious in reference 51. In reference 52, the name of journal is missing. Still the statistical significance is not discussed.

**Response from author:** Revised as suggested. Reference 51 has been replaced with a more appropriate citation revealing the cutoff value for drug discovery. The journal name of reference 52 has been added.

22.	The key amino acid residues in case of F0 receptor are all different for 3 ligands. How do these compare with that of chloroquine? Does the dissimilarity point to the same pocket? Please provide additional interpretation.

**Response from author:** It is the same with chloroquine as shown in Figures 2 and 4. Binding domains on the receptor proteins are deferent, with the exception for hMPV

23.	The same point as in number 21 applies to another receptor G.

**Response from author:** Revised as suggested.

24.	The scores for chloroquine are not better than -6 kcal/mol in two cases. Please comment.

**Response from author:** docking energy scores are presented by Autodock Vina software that estimates the binding affinity between a ligand and a protein target.

25.	LYS-254 is the only common one in case of hMPV. Please compare with those with chloroquine. The type of interaction is not disclosed.

**Response from author:** It is the same with chloroquine as shown in Figures 4. Binding domain on the hMPV receptor protein is the same.

26.	Chloroquine showed AMES toxicity, hepatotoxicity, hERG II inhibition, and low HIA. It was predicted to be a substrate for 4 instances. The ADMET prediction was not suitable for it to be a safe drug. Please clarify.

**Response from author:** ADMET for chloroquine was performed to compare with digoxigenin, cedrene and cedrol since chloroquine is the control used in the experiment. The goal is to show the potential safety of digoxigenin, cedrene and cedrol besides their possible antiviral potential. Certain antimicrobial agents can have AMES toxicity, hepatotoxicity, hERG II inhibition, but if taken in large doses.

27.	“RMSD and RMSF values of protein-ligand complexes were compared to identify any minor or major alterations in the structural configuration of viral protein receptor after ligand binding.” Compared with which other case? The results before ligand binding are not reported.

**Response from author: “**compared” changed to “monitored” in the statement.

28.	“Lower RMSD value has been proposed as an indicator of more stable docked configuration [56].” This reference discusses about crystal structures with good resolution. The proteins in this study have high values. Please correlate the current case with that mentioned in the reference.

**Response from author:** Revised as suggested. Reference 56 has been replaced with a more appropriate citation revealing the significance of lower RMSD.

29.	Table 5 may be better with the results of chloroquine for better comparison. The use of multiple chains is not clear. Please clarify and provide adequate references.

**Response from author:** NiV attachment glycoprotein (G) (PDB ID: 2VWD) consist of two chains in its protein structure file (Chain A and Chain B), Meanwhile, the NiV fusion glycoprotein (F0) (PDB ID: 5EVM) has six chains in its protein structure file (Chain A, Chain B, Chain C, Chain D, Chain E and Chain F). In which, they are differ from each other. All these chains individually interact with the selected ligand upon docking with per-residue analysis and therefore, it is important to show their MD simulations.

30.	The calculation of the apo form of the proteins for relative stability analysis would help readers to assess the consequences of adduct formation. A significant point seems to be missing.

**Response from author:** The protein backbone or "Apo protein simulation has been added.

31.	Please adjust the significant figures for distances.

**Response from author:** Revised as suggested.

32.	“In case of 2VWD, all ligands molecules provide protein-like stability.” Please elaborate.

**Response from author:** corrected from 2VWD to 5WB0. Corrected from 2VWD to 5WB0. Sentence meaning improved to “In case of 5WB0, all ligands molecules maintained consistent protein stability throughout RMSD evaluation”.

33.	“Subsequently, minor fluctuations between 250 and 300 residues were observed for 5WB0 and its ligand complexes” Where are these residues located and are there any disruption to ligand binding?

**Response from author:** These residues are located on PDB ID: 5WB0 (hMPV fusion glycoprotein F0). A minor disruption to the ligand binding maybe evident.

34.	“Whereas chloroquine is one of the very few potential agents that has been proposed as an option against preventing the host-cell attachment and entry of NiV [66]” This reference does not include chloroquine. Please provide an adequate one. Also, the author names are missing in the list.

**Response from author:** Revised as suggested. Citations have been corrected.

35.	“We selected four compounds of plant origin for which data in the literature have demonstrated antiviral properties against different viruses”. Please add the references.

**Response from author:** Revised as suggested.

36.	Lines 502 and 503 are broken. Please fix it. It is better in introduction section where a case is built.

**Response from author:** lines 502 and 503 paragraphically separates the data for RSD and RMSF results.

37.	Relevance of reference 73. Please elaborate.

**Response from author:** reference and sentence removed “Despite showing a weak viral protein interaction in our study, other investigations have indicated that lauric acid have induced antiviral activity against the vesicular stomatitis virus by preventing the binding of viral M protein with host cells”.

38.	Lines 504-523. If the ligands have been reported to possess antiviral properties in the literature, then in silico could be carried out directly for theoretical assessment. The source may be optional. The objective of exploration of natural product-based formulation from the 3 plants chosen could be mentioned.

**Response from author:** The objective has been already mentioned at the beginning of the abstract and at the end of the introduction.

39.	“ADME and toxicity studies of chloroquine, digoxigenin, cedrene and cedrol has been previously reported with slight variations in analysis parameters” What are the slight variations and are they relevant?

**Response from author:** Sentence revised and “with slight variations in analysis parameters” removed.

40.	“MD simulations showed that when the drug receptor complex's structure changed over time, it stabilized in the physiochemical environment.” How is this claim derived?

**Response from author:** Molecular dynamics (MD) simulations are capable of illustrating the structural alterations of drug-receptor complexes, and a sustained complex over time, typically signified by minimal root-mean-square deviation (RMSD) or fluctuations, indicates beneficial interactions and stability within the physicochemical environment. This stability is essential for the effectiveness of the drug, as it suggests that the complex upholds its structural integrity and anticipated binding mode in physiological conditions, thereby aiding in the validation of computational models designed for drug development.

41.	Please avoid the phrase virtual screening since only 4 phytochemicals were used. Also, it is not ADMET-AI. Please omit it (AI) from keywords and abstract.

**Response from author:** Revised as suggested. “Virtual screening” has been replaced by “*in silico* analysis”

42.	Optional. Other parameters like gyradius, SASA, and radial pair distribution function would help to better assess the level of adduct stability.

**Response from author:** Radius of gyration, SASA, H-bonds has been added.

Considering all the points, it seems obvious that the manuscript is not suitable for publication in its current form. A major revision with correct, clear, and acceptable answers would likely render it for the next stage.

Thank you.

Sincerely,

Reviewer

Reviewer’s Comment

Manuscript Number: PONE-D-25-23763

Title: Major secondary metabolites present in Cleistanthus bracteosus Jabl. and essential oils of Artemisia scoparia and Thuja orientalis have anti-Nipah virus and anti-human metapneumovirus potential: An AI-driven in-silico study

Review Summary

This study deals with the investigation of anti-Nipah virus and anti-human metapneumovirus potential: of major secondary metabolites present in Cleistanthus bracteosus Jabl. and essential oils of Artemisia scoparia and Thuja orientalis. This present study offers computational insights into the nti-Nipah virus and anti-human metapneumovirus potential of these metabolites by using an AI-driven in silico approach. The binding affinities further revealed that some of these compounds interacted more strongly with NiV and hMPV receptors than the drug control chloroquine (-5.5 to -6.1 kcal/mol). Moreover, MD simulations illustrated phytochemical interacting amino acid residues associated with each receptor of NiV and hMPV. These phytochemical compounds were further evaluated using ADMET-AI platforms. The research is relevant, however, several aspects of the study, particularly in terms of methodological clarity and scientific rigor, require significant improvement for the manuscript to meet publication standards.

•	The keywords are too long and too many. It is recommended to limit the keywords between four to six

**Response from author:** Revised as suggested.

•	Line 70, there should be a comma after the word “particular”

**Response from author:** Revised as suggested.

•	Below line 186 under the materials and method, authors should comment on the grade of reagents and solvents used or report on the purity level of these reagents and solvents

**Response from author:** Revised as suggested.

•	Line 215, kindly justify why extraction temperature as high as 100oC was used knowing fully well that most of the bioactive metabolites are heat-labile

**Response from author:** maintaining temperatures between 90°C and 100°C is the standard protocol for the extraction of essential oil via steam distillation. Bioactive metabolites in the essential oils in our experiment did not degrade or disintegrate.

•	Line 219, the description of the rotary evaporation should the phrase “under reduced pressure”

**Response from author:** Revised as suggested.

•	Line 494: put the expression “in vitro” in italics

**Response from author:** Revised as suggested.

•	Line 543, the word activity should be in plural form to become “anti-NiV and anti-hMPV activities”

**Response from author:** Revised as suggested.

•	Line544, there should be a comma after the word “particularly”

**Response from author:** Revised as suggested.

•	Line 778 Ref 67: put the expression “in vivo” in italics

**Response from author:** Revised as suggested.

•	Line 789 Ref 71: put the expression “in vitro” in italics

**Response from author:** Revised as suggested.

•	Line 826 Ref 84: put the expression “in vitro” in italics

**Response from author:** Revised as suggested.

•	Line 606 Ref 2, the article title should be in lower case to become “Pandemics throughout history”

**Response from author:** Revised as suggested.

•	Line 617 Ref 8, the article title should be in lower case to become “Virus transmission from bats to humans associated with drinking traditional liquor made from Date Palm Sap, Bangladesh”

**Response from author:** Revised as suggested.

•	Line 675 Ref 28, kindly put the name “Cleistanthus collinus” in italics being a botanical name.

**Response from author:** Revised as suggested.

•	Line 677 Ref 29: kindly put the name “Artemisia scoparia” in italics being a botanical name.

**Response from author:** Revised as suggested.

•	Line 683 Ref 32: the article title should be in lower case to become “Phytochemical 684 profiling, antioxidant, antimicrobial and cholinesterase inhibitory effects of essential 685 oils isolated from the leaves of Artemisia scoparia and Artemisia absinthium

**Response from author:** Revised as suggested.

•	Line 685 Ref 32: kindly put the names “Artemisia scoparia” and “Artemisia absinthium” in italics being botanical names.

**Response from author:** Revised as suggested.

**REVIEWER 2 COMMENTS AND RESPONSES**

**Main response from first author:**

Thank you for your valuable comments which helped us to make significant improvements to this manuscript. We have made the suggested amendments that you have requested where possible.

All suggested revisions and corrections made according to Reviewer 2 have been highlighted in green colour in the manuscript.

In case where all reviewers have made the same/similar request/s, those responses have

been highlighted in teal colour in the manuscript.

**Reviewer #2:** This manuscript presents an in silico study investigating the potential antiviral activity of compounds from Cleistanthus bracteosus, Artemisia scoparia, and Thuja orientalis against Nipah virus (NiV) and human metapneumovirus (hMPV). The study utilizes GC-MS to identify major compounds, followed by molecular docking and molecular dynamics simulations to assess binding interactions with NiV and hMPV glycoproteins, and ADMET-AI analysis for pharmacokinetic and toxicity predictions. The findings suggest that digoxigenin, cedrene, and cedrol exhibit promising binding affinities to the viral targets.

While the study addresses an important public health issue (NiV and hMPV outbreaks) and explores the potential of natural products, which are relevant given the lack of approved treatments for NiV and limited treatment for hMPV, the manuscript requires significant revisions before it can be considered for publication.

The primary concern is the overstatement of "anti-viral potential" based solely on in silico results without any experimental validation.

The methodologies, particularly regarding ligand selection and MD simulation analysis, also need greater clarity.

The abstract and conclusion should more accurately reflect the predictive nature and limitations of the in silico approach.

The title, abstract, introduction, results, and conclusion frequently use terms like "anti-Nipah virus and anti-human metapneumovirus potential" and suggest the compounds "have anti-Nipah virus and anti-human metapneumovirus potential" or "can be considered potential antiviral agents". These are strong claims based purely on computational predictions (binding affinity and predicted ADMET/toxicity profiles). In silico studies provide predictions and hypotheses, but they do not demonstrate actual antiviral activity. This requires experimental validation (e.g., in vitro cell culture assays). The language throughout the manuscript must be revised to accurately reflect that the study predicts or suggests potential activity based on computational modeling, emphasizing the need for future experimental validation. The title should be revised to reflect the predictive or in-silico nature of the findings more explicitly. Phrases like "predicted potential" or "in-silico analysis suggests potential" would be more appropriate.

**Response from author:** Revised as suggested. Corrected to “predicted potential”

Justify the rationale for selecting the four specific ligands (digoxigenin, lauric acid, cedrene, cedrol) is primarily based on their "high abundance" or being "major compounds" in the plant extracts, and "prominence of inducing a variety of broad-spectrum antimicrobial activities.

The GC-MS results list percentage abundances: cedrene (11.627%), cedrol (13.487%), digoxigenin (4.178%), and lauric acid (0.769%). While cedrene and cedrol are the most abundant in their respective essential oils, digoxigenin is only 4.178% and lauric acid a mere 0.769% in the *C. bracteosus* fraction. Justifying the selection of lauric acid based on "high abundance" appears inaccurate according to the provided data.

**Response from author:** Digoxigenin and lauric acid were the only phytochemical compounds detected in the most bioactive TLC isolated fraction of *C. bracteosus*. Unlike cedrene from *A. scorparia* and cedrol from *T. orientalis*, they were not selected based on abundance. However, the total area percentage for lauric acid is 2.936% (0.769% from bark fraction 6 plus 2.167% from bark fraction 7). You may refer to the supplementary table added that represents the full GC-MS data of *C. bracteosus* chloroform purified fractions (Table S1) and *A. scorparia* and *T. orientalis* (Table S2) for more details.

The connection between "broad-spectrum antimicrobial activities" and antiviral activity against specific viruses like NiV and hMPV needs to be clearly explained or justified. While some compounds may have both, the mechanisms are often different.

**Response from author:** broad spectrum referred to as these three plants having a range of antimicrobial activities against different pathogens (e.g. antibacterial, antifungal, antiviral, etc.). Based on our primary research and other studies. More details of similar prior antiviral studies of *C. bracteosus*, *A. scorparia* and *T. orientalis* and their potential use as therapeutic agents in folklore medicine to treat viral like infections has been added.

Reference is made to previous studies on antimicrobial/antiviral properties, but the specific relevance of these particular compounds' previously known activities to the chosen viral targets and mechanisms (attachment and fusion glycoproteins) should be explored to strengthen the selection rationale.

**Response from author:** There are no direct antiviral studies related to the selected compounds. However, I have added a sentence to show the significance of essential oil compounds (e.g. cedrol and cedrene are EO compounds) and digoxigenin interacting with viral surface proteins like glycoproteins.

Figure 1, mentioned as showing the GC-MS chromatograms, is not very clear and uninterpretable. Without the proper figure, it is difficult to fully appreciate the basis for identifying these specific compounds as "major" or understanding their relative abundance profiles. While the percentages are given in the text, enhancing the visualizing of the chromatograms would be helpful.

**Response from author:** Kindly refer to the uploaded images (PNG files) in the journal platform, which are of high resolution and not the images present in the converted PDF. Images are needed to be directly added to the PDF upon acceptance for publication.

While binding affinities are provided for all tested ligands and targets, the interacting amino acid residues are only listed for digoxigenin, cedrene, and cedrol, not lauric acid or chloroquine. Including the interactions for lauric acid would complete the docking picture for all test ligands. Including those for the control (chloroquine) would allow for a comparison of how the experimental ligands interact with the targets relative to the reference drug.

**Response from author:** Lauric acid and chloroquine (control) docking images added to figures.

Figures 2, 3, and 4 are described as showing 3D visualizations of molecular interactions, binding domains, binding sites, and interacting amino acids. These figures are crucial for visualizing the docking results and should be included. The text describes binding domains/sites (a, b) and interacting amino acids (c) for each complex, but the descriptions are general. More specific details on the nature of the interactions (hydrogen bonds, hydrophobic interactions, etc.) derived from the docking analysis would provide deeper insight into the binding mechanisms.

**Response from author:** specific details on the nature of the interactions (hydrogen bonds, hydrophobic interactions) and their interacting amino acid residues with MD simulations has been added.

MD simulations were performed for compounds with binding affinities ≤ -6 kcal/mol. This threshold seems appropriate given the range of reported affinities.

The RMSD and RMSF analyses are presented with numerous specific chain identifiers (e.g., Chain E, Chain A, Chain B, Chain D, Chain F, Chain C). It is unclear what these chains represent within the context of the viral glycoproteins (e.g., are they different subunits, different monomers in a multimeric structure, or artifacts of the PDB file structure?). This needs clarification. Without this context, the specific peaks and average values reported for different chains are difficult to interpret.

**Response from author:** NiV attachment glycoprotein (G) (PDB ID: 2VWD) consist of two chains in its protein structure file (Chain A and Chain B), Meanwhile, the NiV fusion glycoprotein (F0) (PDB ID: 5EVM) has six chains in its protein structure file (Chain A, Chain B, Chain C, Chain D, Chain E and Chain F). In which, they are differ from each other. All these chains individually interact with the selected ligand upon docking with per-residue analysis and therefore, it is important to show their MD simulations.

The interpretation of the RMSD and RMSF results in the text appears somewhat contradictory. The abstract mentions "fluctuations at various points" but "remain mostly stable afterwards till 100 ns". The results section states that some chains showed "highest RMSD peak" at specific times but then discusses overall stability, like "remain around 0.15-0.3 nm without significant fluctuations till 100 ns" for 2VWD complexes. A more consistent interpretation of the simulation results, possibly referencing the specific regions of fluctuations (as implied by trajectory analysis), is needed.

The correlation between specific structural changes (beta-turn-beta and helix-coil-helix alterations) mentioned in the abstract and the observed RMSD/RMSF fluctuations should be elaborated upon in the results and discussion.

**Response from author:** RMSD fluctuations are more obvious up to 50 ns and seemingly getting stable between 50 ns and 100 ns. Structural alterations usually occur in beta-turn-beta and helix-coil-helix in proteins. It is a generalized statement (reference added).

Tables 3 and 4 present a wealth of ADMET and toxicity data. The discussion highlights several points, such as human tolerance, T. pyriformis toxicity, skin permeability, BBB/CNS permeability, Caco-2 permeability, P-glycoprotein, CYP enzymes, AMES toxicity, and hERG inhibition, often comparing test ligands to chloroquine. While a general comparison is made, a more in-depth interpretation of what these properties mean for potential drug development is necessary. For example, "higher skin permeability than -2.5 log Kp" is stated, but the significance of this value and whether it is considered high or low relative to desirable drug properties should be clarified.

Similarly, the significance of T. pyriformis toxicity (> -0.5 log ug/L) and Minnow toxicity (> 0.246 log mM) in the context of human drug safety needs explanation.

**Response from author:** already have mentioned about the significance by referring to PkCSM toxicity endpoints. These endpoints show upper/lower thresholds for drug candidates. Also, the sentence clarity has been further improved to show this significance by adding “for therapeutic drug candidates”.

The statement that digoxigenin, cedrene, and cedrol "showed outstanding characteristics that were potentially beneficial during a clinical trial, since they did not indicate AMES toxicity" is a strong claim. While avoiding AMES toxicity and hERG inhibition are desirable properties, predicting "beneficial characteristics during a clinical trial" solely from these in silico predictions is speculative and should be revised.

**Response from author:** The sentence has been revised by changing to “showed adequate characteristics that can be potentially beneficial to qualify as safe antiviral agents”.

Minor comments

The introduction provides background on NiV and hMPV. The discussion of hMPV classification (A1, A2a, A2b, B1, B2, with gene duplication variants A2b1, A2b2, A2c) and the need for nomenclature reassessment is detailed but the relevance of this genotypic diversity to the chosen hMPV target protein (fusion glycoprotein F0, PDB ID 5WB0) is not explicitly stated. Is PDB ID 5WB0 representative of a specific hMPV subtype, and does this classification impact the generalizability of the findings across different hMPV strains?

**Response from author:** It is just to represent the diversity of variants of these viruses and what is more to be discovered about them. However, their structural composition, such as surface proteins are the same even among their respective variants.

The discussion could benefit from a more critical evaluation of the in silico methods used and their limitations. While it mentions that in silico methods are low-cost and safe for screening, it should also acknowledge that these are predictive tools and the results are theoretical until experimentally validated.

**Response from author:** Revised as suggested.

The comparison of findings with previous studies is good, but linking the previously reported activities of these compounds (or related ones) to the current study's in silico findings against NiV/hMPV targets could be more direct and analytical.

**Response from author:** There are no direct antiviral studies related to the selected compounds. However, I have added a sentence to show the significance of essential oil compounds (e.g. cedrol and cedrene are EO compounds) and digoxigenin interacting with viral surface proteins like glycoproteins.

There appear to be some formatting inconsistencies and potential typos (e.g., line breaks mid-sentence in some sections, "weas" instead of "was" in RMSF description). These should be corrected during revision.

**Response from author:** Revised as suggested.

Ensure all statements drawn from sources are cited. For example, the statement in the abstract "The three out of four compounds tested exhibited remarkable binding affinities..." and the specific affinity values should be cited.

**Response from author:** Revised as suggested.

**REVIEWER 3 COMMENTS AND RESPONSES**

Main response from first author:

Thank you for your valuable comments which helped us to make significant improvements to this manuscript. We have made the suggested amendments that you have requested where possible.

All suggested revisions and corrections made according to Reviewer 2 have been highlighted in yellow colour in the manuscript.

In case where all reviewers have made the same/similar request/s, those responses have

been highlighted in teal colour in the manuscript.

Reviewer #3: The manuscript lacks the scientific strength or depth and rigor and is not up to the standards for publishing in PLOS One. However, the manuscript can be improved and following points need to be addressed –

1. INTRODUCTION

I. In introduction line 111 and 132-133 are basically repetition. Please avoid repeating the same info. Revise line 160.

**Response from author:** Figure added as suggested.

II. A genomic organization and structure of the virus is highly recommended.

**Response from author:** Figure added as suggested.

III. While literature about the viral background is detailed, the link to drug discovery is somewhat lacking.

**Response from author:** Revised as suggested. More details linking to antiviral drug discover has been added.

IV. Justification of selecting these 3 plants are not well contextualized. Why do the authors think that these plants would give antiviral activity or have potential antiviral activity particularly against these 2 viruses? These have to be mentioned in the introduction with suitable references.

**Response from author:** Revised as suggested. More details of similar prior antiviral studies of *C. bracteosus*, *A. scorparia* and *T. orientalis* and their potential use as therapeutic agents in folklore medicine to treat viral like infections has been added. However, direct studies involving these three plants against NiV and hMPV has not yet been published. Hence, this can be considered as a pioneering study to show the significance of antiviral potential against via NiV and hMPV *in* *silico* methods. These three plant specimens were a part of our primary research focusing on antimicrobial and synergistic activities.

V. The discussion on the evolution of hMPV should be very brief. It tends to distract the focus of the study objective. Rather more information on the traditional, pharmacological and phytochemicals of the plant would have been better match with the scope of this study.

**Response from author:** Revised as suggested.

VI. I am not sure whether the term ‘broad spectrum’ is appropriate to use here. Why the authors would claim as broad spectrum?

**Response from author:** broad spectrum referred to as these three plants having a range of antimicrobial activities against different pathogens (e.g. antibacterial, antifungal, antiviral, etc.). Based on our primary research and other studies.

VII. The objectives could be more clear and specific.

**Response from author:** Revised as suggested.

2. Methodology

I. How the bioactivity of the TLC fraction were obtained? The solvents used for extraction and fractionation are both toxic and how this fractions are clinically significant in practical application?

**Response from author**: These three plant specimens were a part of our primary research focusing on antimicrobial and synergistic activities. In case of *C. bracteosus*, the title” Chemical profiling and evaluation of *in vitro* antibacterial and antibiotic synergistic activities of *Cleistanthus bracteosus* Jabl. from Malaysian rainforests” is under consideration on another journal, which houses most of the data related to *C. bracteosus.* That work demonstrated how the bioactivity of the TLC fraction of was *C. bracteosus* obtained. The aforesaid title shall be cited in this study if published in advanced. Solvent toxicity needs be discussed during *in vitro*, *in vivo* or *ex vivo* settings and not in silico. In silico studies only promote the test agent for the aforesaid studies based on compatibility and efficiency.

II. What drove the authors to analyse the phytochemicals by GC-MS since it will provide the information about non-polar, volatile compounds. Is there any evidence or reports that directed to focus on these types of compounds?

**Response from author**: GC-MS data were gathered from our primary research based on these three plants. Meanwhile, digoxigenin and lauric acid were the only phytochemical compounds detected in the most bioactive TLC isolated fraction of *C. bracteosus*. Unlike cedrene from *A. scorparia* and cedrol from *T. orientalis*, they were not selected based on abundance. However, the total area percentage for lauric acid is 2.936% (0.769% from bark fraction 6 plus 2.167% from bark fraction 7). You may refer to the supplementary table added that represents the full GC-MS data of *C. bracteosus* chloroform purified fractions (Table S1) and *A. scorparia* and *T. orientalis* (Table S2) for more details.

III. The docking experiments should be validated. No reference compound/drug was included to compare with. This is important to understand the possible anti viral activity of these phytocompounds.

**Response from author:** chloroquine is the reference drug, which has been already included.

IV. Did the author included the co-crystalized ligand in the docking? It would help in validating the docking work. Moreover, the compounds bound to the amino acid residues at the binding pocket but it is not discussed how significant are these residues. Are these residues important for the activity?

3. Results

I. The quality of the figures is not good. Should provide good resolution images.

**Response from author:** Kindly refer to the uploaded images (PNG files) in the journal platform, which are of high resolution and not the images present in the converted PDF. Images are needed to be directly added to the PDF upon acceptance for publication.

II. For the MD simulation, it is important to add calculations for more parameters like, MMBSA/MMPBSA, radius of gyration, hydrogen bonding, PCA analysis to understand the binding energy convergence, or conformational dynamics of the ligand-protein complexes.

**Response from author:** Revised as suggested. All those added except for PCA since its laborious, and MMPBSA is already telling us that almost all candidates (except Cedrol + 2VWD) presented excellent or very good binding energies and chloroquine did not do well.

III. Why the authors did not provide RMDS and RMSF data for the apo protein? Inclusion of reference drug is also important to compare the binding stability for these compounds and understand the potential activity.

**Response from author:** The protein backbone or "Apo protein simulation has been added.

**REVIEWER 4 COMMENTS AND RESPONSES**

Main response from first author: None requested.

**REVIEWER 5 COMMENTS AND RESPONSES**

Thank you for your valuable comments which helped us to make significant improvements to this manuscript. We have made the suggested amendments that you have requested where possible.

All suggested revisions and corrections made according to Reviewer 2 have been highlighted in turquoise colour in the manuscript.

In case where all reviewers have made the same/similar request/s, those responses have

been highlighted in teal colour in the manuscript.

Reviewer #5: This manuscript presents an in silico study using AI-driven molecular docking, molecular dynamics simulations, and ADMET predictions to evaluate the antiviral potential of major phytochemicals from Cleistanthus bracteosus, and essential oils from Artemisia scoparia and Thuja orientalis against Nipah virus (NiV) and human metapneumovirus (hMPV). The study identifies several compounds with higher predicted binding affinities than chloroquine, a known antiviral agent, and discusses their interaction profiles and predicted pharmacokinetic properties. However, I have some reservations listed below:

1. The study is entirely in silico. While computational predictions are valuable for hypothesis generation, the absence of any in vitro or in vivo validation limits the immediate impact and practical applicability of the findings.

**Response from author:** Unfortunately, we do not have the funds, authorization or facilities to conduct *in vitro* or *in vivo* experimentation for Nipah virus (NiV) and human metapneumovirus (hMPV). Hence, the potential antiviral therapy against NiV and hMPV has been hypothetically drawn via in silico methods, in order to deliver the idea and recommendation for conducting actual research under *in vitro*, *in vivo* or *ex vivo* settings for future studies.

2. Only a small number of compounds (four) were selected for detailed docking and simulation, which may not fully represent the antiviral potential of the source plants and oils.

**Response from author:** We tested the most significant compounds present in the extracts of *Cleistanthus bracteosus*, and essential oils from *Artemisia scoparia* and *Thuja orientalis* based on their quantitative abundance and antimicrobial potential from the primary mesarch.

3. The manuscript references "AI-driven" methods (e.g., ADMET-AI), but the specific AI models, their validation, and their performance metrics are not described in detail, which may limit reproducibility and assessment of the robustness of the predictions.

**Response from author:** The AI model has been added (Chemprop-RDKit model). PkCSM pharmacokinetics analyses ADMET via automated algorithms and artificial intelligence.

4. The abstract and conclusion suggest these compounds could be used as novel therapeutic strategies, but without experimental validation, such statements should be more cautiously framed.

**Response from author:** The term “potential” has been used, which does not clarify or confirm these compounds to be qualified as novel therapeutic agents for the selected viruses.

5. There are minor grammatical errors and awkward phrasings throughout the abstract and initial sections, which may hinder readability and should be addressed in revision.

**Response from author:** Revised as suggested.

6. Chloroquine is used as a control, but its relevance as a comparator for NiV and hMPV (for which it is not a standard therapy) should be justified or alternative controls considered.

**Response from author:** Research utilizing animal models has also investigated the potential application of chloroquine against NiV and hMPV, where chloroquine has been used as an antagonist in preventing the host-cell attachment and entry of these two viruses. However, currently there is no standard antiviral therapy for these viral infections. Additional references have been added to justify this statement.

**REVIEWER 6 COMMENTS AND RESPONSES**

Thank you for your valuable comments which helped us to make significant improvements to this manuscript. We have made the suggested amendments that you have requested where possible.

All suggested revisions and corrections made according to Reviewer 2 have been highlighted in pink colour in the manuscript.

In case where all reviewers have made the same/similar request/s, those responses have

been highlighted in teal colour in the manuscript.

Reviewer #6: Comments for PONE-D-25-23763

I have gone through the assigned manuscript entitled “Major secondary metabolites present in Cleistanthus bracteosus Jabl. and essential oils of Artemisia scoparia and Thuja orientalis have anti-Nipah virus and anti-humanmetapneumovirus potential: An AI-driven in-silico study”, and found that it is competently well-written and the manuscript contains sufficient data, but it should meet the following comments:

Major comments-

1- Title should be shortened.

**Response from author:** Revised as suggested.

2. The abstract should provide a concise summary of the key findings of the whole study.

**Response from author:** Revised as suggested.

3- Authors added all old references in the Introduction, so I suggest adding more recently published work with your relevant studies (2023-2025) and rewriting the Introduction section.

**Response from author:** Revised as suggested.

4- The material and methods should include more details.

**Response from author:** Revised as suggested.

5- The authors have to compare their results with literature data and improve the results and the discussion section completely.

**Response from author:** Revised as suggested. There are hardly any direct studies. Hence, have added and compared with similar studies.

6- Follow the unit in the same system throughout the manuscript.

**Response from author:** Revised as suggested.

7- Why did the author use both NiV and (hMPV proteins for docking studies? Give your appropriate reasons properly and separately. Your explanation should be added to the manuscript's results and discussion sections.

**Response from author:** Both NiV and hMPV are emerging viruses capable of causing global outbreaks, in which several outbreaks have been reported in the recent past from multiple countries. No vaccination or approved antiviral treatments available for managing the infections caused by these two viruses as of yet. Therefore, we believe that it would be prudent to propose a safe potential antiviral therapy against NiV and hMPV via *in silico* methods, in order to deliver the idea and recommendation for conducting actual research under *in vitro*, *in vivo* or *ex vivo* settings for future studies.

8- Mention which docking algorithm and scoring function were utilized in the docking process.

**Response from author:** Revised as suggested. The scoring function for AutoDock Vina was based on 6–12 van der Waals interactions and Coulomb energies, combined with Broyden-Fletcher-Goldfarb-Shanno algorithm. Also, reference added.

9- How do authors validate the protein? Add a Ramachandran plot to illustrate the distribution of phi (φ) and psi (ψ)dihedral angles for the protein's residues.

**Response from author:** Revised as suggested. Ramachandran plot added for 2VWD: NiV attachment glycoprotein (G), 5EVM: NiV fusion glycoprotein (F0) and 5WB0: hMPV fusion glycoprotein (F0) (Figure S1).

10- In the results and discussion, the authors should strengthen the explanation of the validity of the native ligand docking results.

**Response from author:** Revised as suggested.

11- Authors are suggested to add the following articles to the manuscript.

Deleted by editor.

12- References are not properly cited and should follow the journal's style. The References section has to be revised after all corrections.

**Response from author:** Revised as suggested.

13-In the manuscript, there are many spelling and grammatical errors. So, grammatical and punctuation errors must be corrected. I suggest improving the English language.

**Response from author:** Revised as suggested.

14-Conclusion should be succinct and precise, not be same as the Abstract.

My Opinion: Major Revision.

**Response from author:** Revised as suggested.

**REVIEWER 7 COMMENTS AND RESPONSES**

Thank you for your valuable comments which helped us to make significant improvements to this manuscript. We have made the suggested amendments that you have requested where possible.

All suggested revisions and corrections made according to Reviewer 2 have been highlighted in grey colour in the manuscript.

In case where all reviewers have made the same/similar request/s, those responses have

been highlighted in teal colour in the manuscript.

Reviewer #7: Reviewer’s Comment

Manuscript Number: PONE-D-25-23763

Title: Major secondary metabolites present in Cleistanthus bracteosus Jabl. and essential oils of Artemisia scopariaand Thuja orientalis have anti-Nipah virus and anti-human metapneumovirus potential: An AI-driven in-silico study

Review Summary

This study deals with the investigation of anti-Nipah virus and anti-human metapneumovirus potential: of major secondary metabolites present in Cleistanthus bracteosus Jabl. and essential oils of Artemisia scoparia and Thuja orientalis. This present study offers computational insights into the anti-Nipah virus and anti-human metapneumovirus potential of these metabolites by using an AI-driven in silico approach. The binding affinities further revealed that some of these compounds interacted more strongly with NiV and hMPV receptors than the drug control chloroquine (-5.5 to -6.1 kcal/mol). Moreover, MD simulations illustrated phytochemical interacting amino acid residues associated with each receptor of NiV and hMPV. These phytochemical compounds were further evaluated using ADMET-AI platforms. The research is relevant, however, several aspects of the study, particularly in terms of methodological clarity and scientific rigor, require significant improvement for the manuscript to meet publication standards.

• The keywords are too long and too many. It is recommended to limit the keywords between four to six

**Response from author:** Revised as suggested. Keywords reduced to seven.

• Line 70, there should be a comma after the word “particular”

**Response from author:** Revised as suggested.

• Below line 186 under the materials and method, authors should comment on the grade of reagents and solvents used or report on the purity level of these reagents and solvents

**Response from author:** Revised as suggested.

• Line 215, kindly justify why extraction temperature as high as 100oC was used knowing fully well that most of the bioactive metabolites are heat-labile

**Response from author:** maintaining temperatures between 90°C and 100°C is the standard protocol for the extraction of essential oil via steam distillation. Bioactive metabolites in the essential oils in our experiment did not degrade or disintegrate.

• Line 219, the description of the rotary evaporation should the phrase “under reduced pressure”

**Response from author:** Revised as suggested.

• Line 494: put the expression “in vitro” in italics

**Response from author:** Revised as suggested.

• Line 543, the word activity should be in plural form to become “anti-NiV and anti-hMPV activities”

**Response from author:** Revised as suggested.

• Line544, there should be a comma after the word “particularly”

**Response from author:** Revised as suggested.

• Line 778 Ref 67: put the expression “in vivo” in italics

**Response from author:** Revised as suggested.

• Line 789 Ref 71: put the expression “in vitro” in italics

**Response from author:** Revised as suggested.

• Line 826 Ref 84: put the expression “in vitro” in italics

**Response from author:** Revised as suggested.

• Line 606 Ref 2, the article title should be in lower case to become “Pandemics throughout history”

**Response from author:** Revised as suggested.

• Line 617 Ref 8, the article title should be in lower case to become “Virus transmission from bats to humansassociated with drinking traditional liquor made from Date Palm Sap, Bangladesh”

**Response from author:** Revised as suggested.

• Line 675 Ref 28, kindly put the name “Cleistanthus collinus” in italics being a botanical name.

**Response from author:** Revised as suggested.

• Line 677 Ref 29: kindly put the name “Artemisia scoparia” in italics being a botanical name.

**Response from author:** Revised as suggested.

• Line 683 Ref 32: the article title should be in lower case to become “Phytochemical 684 profiling, antioxidant, antimicrobial and cholinesterase inhibitory effects of essential 685 oils isolated from the leaves of Artemisia scoparia and Artemisia absinthium

**Response from author:** Revised as suggested.

• Line 685 Ref 32: kindly put the names “Artemisia scoparia” and “Artemisia absinthium” in italics being botanical names.

**Response from author:** Revised as suggested.
