# Supplementary material for: Predicted antiviral potential of phytochemicals prolific in Cleistanthus bracteosus Jabl. and essential oils of Artemisia scoparia and Thuja orientalis against Nipah virus and Human metapneumovirus: An AI-driven in-silico study
Source: PLoS One. 2026 Mar 31;21(3):e0346254. doi: 10.1371/journal.pone.0346254 (PMC13038001; doi:10.1371/journal.pone.0346254)
Supplement: Attachment — Submitted filename: Reviewers Comment for PONE-D-25-023763R1.docx [file pone.0346254.s007.docx]

**Reviewer’s Comment**

**Manuscript Number:** PONE-D-25-023763R1

**Title:** Predicted antiviral potential of phytochemicals prolific in *Cleistanthus bracteosus* Jabl.

and essential oils of *Artemisia scoparia* and *Thuja orientalis* against Nipah virus and

Human metapneumovirus: An AI-driven in-silico study

**Review Summary**

This study deals with the utilization of major compounds present in *Cleistanthus bracteosus* and in the essential oils extracted from *Artemisia scoparia* and *Thuja orientalis* to analyse their antiviral activity against the NiV and hMPV through computational approach. Molecular docking and molecular dynamics simulations were the primary tools used by these authors to assess the binding interactions of the major compounds detected by GC-MS analysis. These phytochemical compounds were further evaluated using ADMET platforms. The authors also reported that RMSD graphs show fluctuations at various points in the protein-ligand complex system due to structural changes in the beta-turn-beta and helix-coil-helix alterations at specific points resulting in increased RMSD within a time frame of 50 ns. However, these alterations in the simulation remain mostly stable afterwards till 100 ns.

**Strengths of the Manuscript**

1. **Comprehensive Computational Workflow**:
   The study employs a multi-tiered *in silico* strategy; molecular docking, molecular dynamics simulations, binding free energy calculations (MM/PBSA), and ADMET profiling, which provides a robust and layered assessment of the compounds’ potential. This methodological rigor is consistent with current standards in computational drug discovery.
2. **Relevant and Timely Virological Targets**:
   Nipah virus (NiV) and human metapneumovirus (hMPV) are both significant public health threats with limited therapeutic options. Targeting their entry glycoproteins (G and F) is a rational and promising antiviral strategy, well justified in the introduction.
3. **Use of Control Compound**:
   Inclusion of chloroquine as a reference adds context to the docking scores and ADMET results, allowing readers to gauge the relative promise of the phytochemicals.
4. **Integration of AI and Machine Learning Tools**:
   The use of AI-driven ADMET platforms (e.g., pkCSM, SwissADME) and modern simulation software (GROMACS, AMBER) reflects contemporary, state-of-the-art practices in computational pharmacology.
5. **Detailed Supporting Analyses**:
   The manuscript includes RMSD, RMSF, SASA, Rg, and hydrogen bond analyses over 100 ns MD simulations, which lends credibility to the stability and interaction patterns of the protein–ligand complexes.
6. **Clear Presentation of Data**:
   Results are well-organized in tables and figures, and the discussion contextualizes findings within existing literature on antiviral phytochemicals.
7. **Ethnobotanical Relevance**:
   The selection of *Cleistanthus bracteosus*, *Artemisia scoparia*, and *Thuja orientalis* is supported by traditional use and prior evidence of bioactive properties, adding translational relevance.

**Originality and Novelty**

1. **First Reported Investigation Against NiV and hMPV**:
   To the best of current knowledge, this is the first *in silico* study to specifically evaluate digoxigenin, cedrene, and cedrol, which is done individually or in combination, against the attachment and fusion glycoproteins of both NiV and hMPV. This represents a novel contribution to the field of antiviral phytochemistry.
2. **Cross-Kingdom Phytochemical Exploration**:
   The combination of compounds from three distinct plant sources which are, a flowering plant, a conifer, and a medicinal herb, broadens the chemical diversity examined and highlights the potential of integrative phytochemical sourcing.
3. **AI-Enhanced Predictive Workflow**:
   While molecular docking and MD are common, the explicit incorporation of AI-based ADMET prediction early in the screening pipeline is a forward-looking approach that aligns with modern drug discovery trends.
4. **Hypothesis-Driven Mechanistic Insight**:
   The proposed mechanism (blocking viral entry via glycoprotein interference) is clearly illustrated and supported by docking and dynamics data, offering a testable model for future experimental validation.

**Overall Assessment of the Manuscript**

This manuscript presents a well-structured, technically sound, and novel computational study that meets the standards for publication in a reputable journal. Its strengths lie in:

- The use of a multi-method computational pipeline that validates findings across several layers of analysis.
- The focus on understudied viral targets with high clinical relevance.
- The integration of traditional botanical knowledge with modern AI-driven tools.
- The clear, hypothesis-driven approach that can guide future *in vitro* and *in vivo* studies.

**Suggested Few Amendments for Enhancement**:

- Clarify the % abundance of digoxigenin in *C. bracteosus* (4.178% is noted but considered “major”, brief justification would help).
- Ensure all figure and table references in the text are correctly placed in the final version.
- Consider briefly discussing the limitations of *in silico* predictions in the conclusion to frame the findings appropriately.

**Recommendation: Accept**

The manuscript demonstrates significant originality in its target–compound combinations and employs a rigorous, contemporary computational methodology. It provides compelling preliminary evidence that digoxigenin, cedrene, and cedrol warrant further experimental investigation as potential broad-spectrum antiviral agents. The work is suitable for publication in a peer-reviewed journal, particularly those focusing on virology, computational biology, or natural product drug discovery. The manuscript has the potential to make a valuable contribution to the field. Hence, from my own opinion, the manuscript could be accepted for publication in Plos ONE.
