# Supplementary material for: Predicted antiviral potential of phytochemicals prolific in Cleistanthus bracteosus Jabl. and essential oils of Artemisia scoparia and Thuja orientalis against Nipah virus and Human metapneumovirus: An AI-driven in-silico study
Source: PLoS One. 2026 Mar 31;21(3):e0346254. doi: 10.1371/journal.pone.0346254 (PMC13038001; doi:10.1371/journal.pone.0346254)
Supplement: Attachment — Submitted filename: Round 2 - PLOS One rebuttal and response to reviewers.docx [file pone.0346254.s008.docx]

**REVIEWER 2 COMMENTS AND RESPONSES**

**Main response from first author:**

Thank you for your valuable comments which helped us to make significant improvements to this manuscript. We have made the suggested amendments that you have requested where possible.

All suggested revisions and corrections made according to Reviewer 2 have been highlighted in red colour in the manuscript.

Reviewer #2: RMSD fluctuations are due to structural changes in the beta-turn-beta and helix-coil-helix alterations. Although noted as a "generalized statement", providing explicit evidence (even brief qualitative examples from the trajectory analysis) that these specific structural regions drive the observed RMSD peaks would strengthen the analysis, rather than relying solely on the general reference.

Though author clarified that lauric acid (0.769%) was selected because it was present in the most bioactive TLC isolated fraction, the introduction or results section should explicitly reinforce that its selection overrides the low abundance percentage due to its detected presence in this highly active fraction, rather than being generally considered "high abundance".

**Response from author:** Revised as suggested (lines 386-387).

Just like before concern as multiple reviewers noted that the most of the figures (including the GC-MS chromatograms and MD plots) were difficult to read. Before final acceptance confirmation is needed that the high-resolution PNG/vector graphic files submitted separately to render all details

**Response from author:** All figures have been uploaded as separate high-resolution PNG files throughput submissions. Kindly get assistance from the editorial team if you are having a difficulty in accessing them. I have given additional instructions on how to access them below:

Kindly click on the circled section to download the high-resolution PNG for each figure.

**REVIEWER 5 COMMENTS AND RESPONSES**

**Main response from first author:**

Thank you for your valuable comments which helped us to make significant improvements to this manuscript. We have made the suggested amendments that you have requested where possible.

All suggested revisions and corrections made according to Reviewer 5 have been highlighted in green colour in the manuscript.

Reviewer #5: The authors have addressed the reviewer comments but some needs to be corrected.

1.The manuscript remains longer than necessary, and several results—particularly MD analyses—are repetitive and should be condensed.

**Response from author:** Revised as suggested (lines 476-480).

2.Molecular dynamics results should be presented more integratively, focusing on overall stability trends rather than detailed metric-by-metric narration.

**Response from author:** Revised as suggested (lines 476-480).

3.Claims implying therapeutic efficacy should be moderated to reflect the predictive, in-silico nature of the study.

**Response from author:** Revised as suggested. Sentence improved to reflect the predictive, *in-silico* nature of the study. (lines 433-434). Also, have already been justified in the abstract (line 69) and conclusion (line 668).

4.The ADMET discussion should more clearly acknowledge the limitations and preliminary nature of computational pharmacokinetic predictions.

**Response from author:** Revised as suggested (lines 654-667). The primary goal of this study to show the binding potential between the selected ligand and proteins, ADMET is only a side analysis to show the predictive quality profile.

5.The Abstract would benefit from reduction in length and numerical detail for improved clarity.

**Response from author:** Revised as suggested.

6.Some sections still contain long, complex sentences; a final language polish is recommended.

**Response from author:** Revised as suggested.

7.Conceptual mechanism figures should be explicitly labeled as hypothetical.

**Response from author:** Revised as suggested.

8.Read and cite below papers:

Patel, C. N., & Mall, R. (2025). Repurposing drugs for monkeypox using artificial intelligence. The Scientific Basis of Mpox (Monkeypox), 421-440.

Patel, C. N., Jani, S. P., Kumar, S. P., Modi, K. M., & Kumar, Y. (2022). Computational investigation of natural compounds as potential main protease (Mpro) inhibitors for SARS-CoV-2 virus. Computers in Biology and Medicine, 151, 106318.

Patel, C. N., Mall, R., & Bensmail, H. (2023). AI-driven drug repurposing and binding pose meta dynamics identifies novel targets for monkeypox virus. Journal of Infection and Public Health, 16(5), 799-807.

**Response from author:** Added as suggested

**REVIEWER 7 COMMENTS AND RESPONSES**

Thank you for your valuable comments which helped us to make significant improvements to this manuscript. We have made the suggested amendments that you have requested where possible.

All suggested revisions and corrections made according to Reviewer 7 have been highlighted in turquoise colour in the manuscript.

Reviewer #7: Reviewer’s Comment

Manuscript Number: PONE-D-25-023763R1

Title: Predicted antiviral potential of phytochemicals prolific in Cleistanthus bracteosus Jabl.

and essential oils of Artemisia scoparia and Thuja orientalis against Nipah virus and

Human metapneumovirus: An AI-driven in-silico study

Review Summary

This study deals with the utilization of major compounds present in Cleistanthus bracteosus and in the essential oils extracted from Artemisia scoparia and Thuja orientalis to analyse their antiviral activity against the NiV and hMPV through computational approach. Molecular docking and molecular dynamics simulations were the primary tools used by these authors to assess the binding interactions of the major compounds detected by GC-MS analysis. These phytochemical compounds were further evaluated using ADMET platforms. The authors also reported that RMSD graphs show fluctuations at various points in the protein-ligand complex system due to structural changes in the beta-turn-beta and helix-coil-helix alterations at specific points resulting in increased RMSD within a time frame of 50 ns. However, these alterations in the simulation remain mostly stable afterwards till 100 ns.

Strengths of the Manuscript

1. Comprehensive Computational Workflow:

The study employs a multi-tiered in silico strategy; molecular docking, molecular dynamics simulations, binding free energy calculations (MM/PBSA), and ADMET profiling, which provides a robust and layered assessment of the compounds’ potential. This methodological rigor is consistent with current standards in computational drug discovery.

2. Relevant and Timely Virological Targets:

Nipah virus (NiV) and human metapneumovirus (hMPV) are both significant public health threats with limited therapeutic options. Targeting their entry glycoproteins (G and F) is a rational and promising antiviral strategy, well justified in the introduction.

3. Use of Control Compound:

Inclusion of chloroquine as a reference adds context to the docking scores and ADMET results, allowing readers to gauge the relative promise of the phytochemicals.

4. Integration of AI and Machine Learning Tools:

The use of AI-driven ADMET platforms (e.g., pkCSM, SwissADME) and modern simulation software (GROMACS, AMBER) reflects contemporary, state-of-the-art practices in computational pharmacology.

5. Detailed Supporting Analyses:

The manuscript includes RMSD, RMSF, SASA, Rg, and hydrogen bond analyses over 100 ns MD simulations, which lends credibility to the stability and interaction patterns of the protein–ligand complexes.

6. Clear Presentation of Data:

Results are well-organized in tables and figures, and the discussion contextualizes findings within existing literature on antiviral phytochemicals.

7. Ethnobotanical Relevance:

The selection of Cleistanthus bracteosus, Artemisia scoparia, and Thuja orientalis is supported by traditional use and prior evidence of bioactive properties, adding translational relevance.

Originality and Novelty

1. First Reported Investigation Against NiV and hMPV:

To the best of current knowledge, this is the first in silico study to specifically evaluate digoxigenin, cedrene, and cedrol, which is done individually or in combination, against the attachment and fusion glycoproteins of both NiV and hMPV. This represents a novel contribution to the field of antiviral phytochemistry.

2. Cross-Kingdom Phytochemical Exploration:

The combination of compounds from three distinct plant sources which are, a flowering plant, a conifer, and a medicinal herb, broadens the chemical diversity examined and highlights the potential of integrative phytochemical sourcing.

3. AI-Enhanced Predictive Workflow:

While molecular docking and MD are common, the explicit incorporation of AI-based ADMET prediction early in the screening pipeline is a forward-looking approach that aligns with modern drug discovery trends.

4. Hypothesis-Driven Mechanistic Insight:

The proposed mechanism (blocking viral entry via glycoprotein interference) is clearly illustrated and supported by docking and dynamics data, offering a testable model for future experimental validation.

Overall Assessment of the Manuscript

This manuscript presents a well-structured, technically sound, and novel computational study that meets the standards for publication in a reputable journal. Its strengths lie in:

• The use of a multi-method computational pipeline that validates findings across several layers of analysis.

• The focus on understudied viral targets with high clinical relevance.

• The integration of traditional botanical knowledge with modern AI-driven tools.

• The clear, hypothesis-driven approach that can guide future in vitro and in vivo studies.

Suggested Few Amendments for Enhancement:

• Clarify the % abundance of digoxigenin in C. bracteosus (4.178% is noted but considered “major”, brief justification would help).

**Response from author:** Revised as suggested (lines 386-387).

• Ensure all figure and table references in the text are correctly placed in the final version.

**Response from author:** All in correct order.

• Consider briefly discussing the limitations of in silico predictions in the conclusion to frame the findings appropriately.

**Response from author:** Revised as suggested (lines 693-700).

Recommendation: Accept

The manuscript demonstrates significant originality in its target–compound combinations and employs a rigorous, contemporary computational methodology. It provides compelling preliminary evidence that digoxigenin, cedrene, and cedrol warrant further experimental investigation as potential broad-spectrum antiviral agents. The work is suitable for publication in a peer-reviewed journal, particularly those focusing on virology, computational biology, or natural product drug discovery. The manuscript has the potential to make a valuable contribution to the field. Hence, from my own opinion, the manuscript could be accepted for publication in Plos ONE.
