# Supplementary material for: Predicted antiviral potential of phytochemicals prolific in Cleistanthus bracteosus Jabl. and essential oils of Artemisia scoparia and Thuja orientalis against Nipah virus and Human metapneumovirus: An AI-driven in-silico study
Source: PLoS One. 2026 Mar 31;21(3):e0346254. doi: 10.1371/journal.pone.0346254 (PMC13038001; doi:10.1371/journal.pone.0346254)
Supplement: Attachment — Submitted filename: Reviewers Comment for PONE-D-25-023763R2.docx [file pone.0346254.s009.docx]

**Revised Reviewer’s Comment**

**Manuscript Number:** PONE-D-25-023763R2
**Title:** Predicted antiviral potential of phytochemicals prolific in Cleistanthus bracteosus Jabl. and essential oils of Artemisia scoparia and Thuja orientalis against Nipah virus and Human metapneumovirus: An AI-driven in-silico study

This study employs a comprehensive computational approach to evaluate the antiviral potential of major phytochemicals from Cleistanthus bracteosus, Artemisia scoparia, and Thuja orientalis against Nipah virus (NiV) and human metapneumovirus (hMPV). Utilizing molecular docking, molecular dynamics (MD) simulations, binding free energy calculations (MM/PBSA), and AI-driven ADMET profiling, the authors provide a robust, multi-layered analysis. The manuscript is well-structured, methodologically sound, and addresses a significant gap in the search for therapeutics against these high-priority viral pathogens.

**Strengths of the Manuscript**

1. **Comprehensive Computational Workflow:** The multi-tiered in silico strategy provides a rigorous assessment, aligning with current standards in computational drug discovery.
2. **Relevant and Timely Virological Targets:** The focus on the entry glycoproteins of NiV and hMPV, both significant public health threats with limited treatments, is highly rational and justified.
3. **Use of a Control Compound:** The inclusion of chloroquine as a reference provides valuable context for interpreting docking scores and ADMET results.
4. **Integration of Modern Tools:** The use of AI-driven ADMET platforms and advanced simulation software reflects state-of-the-art practices.
5. **Detailed Supporting Analyses:** Extensive MD simulation analyses (RMSD, RMSF, SASA, Rg, hydrogen bonds) over 100 ns credibly support the stability and interaction patterns of the complexes.
6. **Clear Presentation of Data:** Results are well-organized and effectively supported by tables and figures, with a discussion that appropriately contextualizes the findings.
7. **Ethnobotanical Relevance:** The plant selection is supported by traditional use and prior evidence of bioactivity, adding translational value.

**Originality and Novelty**

1. **First Reported Investigation:** To current knowledge, this is the first in silico study to specifically evaluate digoxigenin, cedrene, and cedrol against the attachment and fusion glycoproteins of both NiV and hMPV.
2. **Cross-Kingdom Phytochemical Exploration:** Examining compounds from three distinct plant sources broadens the chemical diversity investigated.
3. **AI-Enhanced Predictive Workflow:** The explicit incorporation of AI-based ADMET prediction strengthens the screening pipeline.
4. **Hypothesis-Driven Mechanistic Insight:** The proposed mechanism of blocking viral entry is clearly illustrated and well-supported by the computational data.

**Author Revisions and Final Assessment**
The authors have thoughtfully addressed all minor amendments suggested in the previous review:

- They have clarified the justification for classifying digoxigenin (4.178%) as a major compound.
- All figure and table references in the text have been verified and correctly placed.
- A brief discussion on the inherent limitations of in silico predictions has been added to the conclusion, appropriately framing the study's findings.

**Overall Recommendation**
All necessary corrections have been satisfactorily carried out. The manuscript presents a novel, technically robust, and well-executed computational study that makes a valuable contribution to the field. Its strengths in methodology, relevance, and clarity significantly outweigh any inherent limitations of the in silico approach. The work is now suitable for publication and is **recommended for acceptance in its current form.**
