# Supplementary material for: Predicted antiviral potential of phytochemicals prolific in Cleistanthus bracteosus Jabl. and essential oils of Artemisia scoparia and Thuja orientalis against Nipah virus and Human metapneumovirus: An AI-driven in-silico study
Source: PLoS One. 2026 Mar 31;21(3):e0346254. doi: 10.1371/journal.pone.0346254 (PMC13038001; doi:10.1371/journal.pone.0346254)
Supplement: Attachment — Submitted filename: Round 3 - PLOS One rebuttal and response to reviewers.docx [file pone.0346254.s010.docx]

**REVIEWER 5 COMMENTS AND RESPONSES**

**Main response from first author:**

Thank you for your valuable comments which helped us to make significant improvements to this manuscript. We have made the suggested amendments that you have requested where possible.

All suggested revisions and corrections made according to Reviewer 2 have been highlighted in yellow colour in the manuscript.

Reviewer #5: The authors have substantially addressed the reviewer comments, but the revision is not yet final-submission ready. The remaining issues are presentation, tone, and framing, not missing analyses or methodology.

**Response from author: Made corrections to few typos and sentences from lines 265-268. Apart from that, it is difficult to determine the flaws in presentation, tone, and framing as mentioned by Reviewer #5. Since Reviewer #7 and other reviewers have accepted the manuscript in its current form, I would very much appreciate if Reviewer #5 can specify the corrections that needs to be for** presentation, tone, and framing?

REVIEWER 7 COMMENTS AND RESPONSES

Main response from first author: None requested and acceptance in its current form by Reviewer #7.
